# Finite-Aperture Ultrasound Field Reproduction in Coarse-Grid FDTD via Operator-Consistent Source Reduction

**DOI:** 10.3390/bioengineering13091070

**Published:** 2026-09-15

**Authors:** Hui Zhang, Yang Xu, Weicheng Yan, Yun Wu, Xiang Guo, Xiang Zhou, Pengcheng Zhang, Zaituo Li, Hongrui Tan, Junchao Zeng, Wu Qiu, Chao Cai, Mingyue Ding, Ming Yuchi

**Affiliations:** School of Life Science and Technology, Advanced Bio-Medical Imaging Facility, Huazhong University of Science and Technology, Wuhan 430074, China; d202380973@hust.edu.cn (H.Z.); d202380996@hust.edu.cn (Y.X.); ywc@hust.edu.cn (W.Y.); clound.wu@gmail.com (Y.W.); m202472822@hust.edu.cn (X.G.); xiangzhou_@hust.edu.cn (X.Z.); m202472981@hust.edu.cn (P.Z.); lizt28@hust.edu.cn (Z.L.); d202481103@hust.edu.cn (H.T.); d202380995@hust.edu.cn (J.Z.); wuqiu@hust.edu.cn (W.Q.); chriscai@hust.edu.cn (C.C.); myding@hust.edu.cn (M.D.)

**Keywords:** ultrasound computed tomography, finite-difference time-domain, finite-aperture transducer, equivalent source, operator consistency, source reduction, full-waveform inversion

## Abstract

Full-waveform inversion (FWI) typically employs finite-difference time-domain (FDTD) methods to solve the wave equation under the point-source assumption. For computational efficiency, the coarsest grid that satisfies dispersion and stability requirements is generally preferred. However, this grid may not resolve the finite aperture and spatial phase characteristics of a transducer, thereby introducing imaging artifacts and errors. This paper proposes an Operator-consistent reduced source that maps a local fine-grid radiation field to node-specific forcing histories for a prescribed coarse-grid operator. These histories are reused in repeated simulations without online fine-grid computation. On a 0.50 mm grid, the method reduced root-mean-square (RMS) field relative error by 96.4% compared with the Direct coarse-grid aperture source. In controlled FWI with independent observations, the sound-speed root-mean-square error was 2.14 m/s, compared with 4.94 and 5.17 m/s for the Point source and the Direct coarse-grid aperture source, respectively. Hydrophone measurements, blind ring-water validation, and three repeated phantom acquisitions further assessed field reproduction and reconstruction performance. The method enables coarse-grid FDTD simulations to represent the radiated fields of finite-aperture transducers whose device-scale features are not directly resolved by the target grid.

## 1. Introduction

Ultrasound computed tomography (USCT) reconstructs quantitative maps of tissue acoustic properties from transmitted and scattered pressure signals. Full-waveform inversion (FWI) is one approach to this problem. By matching complete received waveforms rather than only travel times or signal envelopes, FWI uses information carried by refraction, diffraction, phase, and amplitude. It has enabled quantitative reconstruction in breast and ring-array imaging [1,2,3,4,5], and numerical and experimental studies have extended the same framework to transcranial imaging [6]. This additional physical information comes at substantial computational cost because many transmit events and model updates require repeated solutions of the forward and adjoint wave equations [7,8,9].

Traditional FWI implementations commonly represent each transmitter as a Point source. Under this assumption, grid spacing is selected mainly to control numerical dispersion and satisfy the stability condition of the wave solver. For computational efficiency, the coarsest grid that meets these propagation requirements is usually preferred. Such an imaging grid may propagate the target frequency band accurately, yet still be too coarse to represent the finite aperture of the physical transducer.

A real transducer is not a single spatial point. Its aperture, acoustic lens, and spatial amplitude and phase distributions jointly determine the emitted wavefront and angular spectrum [10,11,12,13,14]. Replacing this structure with one node, or with several coarse-grid nodes driven by the same waveform, changes near-field interference, first-arrival phase, and directional illumination. The resulting systematic residual can then be absorbed into the material model during inversion [15,16]. Source representation must therefore be considered part of the discrete forward model rather than a waveform specified independently of the propagation operator.

Existing finite-aperture modeling strategies can be divided into three broad groups. The first group uses device-resolved physical models. Spatial impulse-response, Rayleigh-integral, finite-difference, finite-element, and spectral methods can explicitly represent the aperture, material interfaces, and acoustic lens [10,11,12,17,18]. In this context, a fine grid is one whose spacing is determined by device-scale features, rather than by the propagation wavelength alone. This requirement is much more demanding than the coarse-grid criterion. For example, halving the grid spacing in a three-dimensional explicit solver increases the number of spatial nodes by approximately eight; the corresponding stability limit also requires approximately twice as many time steps, so the computational work can increase by about sixteen-fold before implementation overhead is considered. Repeating such a device-resolved model in every forward and adjoint FWI solution is therefore impractical.

The second group estimates an equivalent source from hydrophone or array measurements [15,16,19,20,21,22]. This approach avoids explicit simulation of the complete device, but the estimated source can contain effects from acquisition electronics, timing, receiver sensitivity, coordinate error, and the calibration medium. Its transfer to a different numerical grid or propagation operator is therefore not automatic.

The third group represents the aperture directly on the imaging grid by distributing a common waveform according to aperture overlap, spatial weights, prescribed delays, or focusing laws [23,24]. This approach is efficient and retains the part of the aperture that the coarse grid can resolve, but direct samples or geometric weights are not generally equal to the discrete forcing required by the target wave operator. Source-box and immersive-injection methods have established that distributed forcing near an injection boundary can reproduce a prescribed wavefield [25,26,27,28]. A remaining practical need is a direct procedure that converts a device-resolved finite-aperture field into reusable forcing for a specified coarse-grid operator: geometric aperture weights alone do not account for the action of that operator on the transferred field.

We address this need by formulating fine-to-coarse source transfer as an operator-specific discrete-forcing problem. One local fine physical simulation supplies the reference field, which is converted into spatially localized, node-specific forcing for the prescribed coarse-grid FDTD operator. The resulting source is reused in subsequent simulations without online fine-grid coupling.

Numerical, hydrophone, and blind ring-water experiments evaluate source-field reproduction, while controlled and repeated real-phantom FWI evaluate its effect on reconstruction. The comparison controls and validation design are specified in the Section 2 and Section 3.

This study makes three contributions. (a) It derives an operator-specific mapping from a converged finite-aperture field to spatially localized, node-specific forcing on a prescribed coarse-grid FDTD operator. (b) It evaluates forward-field reproduction using numerical reference fields, hydrophone measurements, and blind full-array water data. (c) It examines how the three source representations affect reconstruction in controlled and real-phantom FWI without assuming that improved source-field agreement must produce the same ranking for every inversion metric.

## 2. Method

### 2.1. Method Overview

The target coarse grid is designed to propagate the frequency band of interest efficiently, but it may not resolve the finite aperture, acoustic lens, and intra-aperture phase distribution of the transducer. We therefore separate source construction from repeated wave propagation. During the offline stage, a local fine physical model is solved around the transducer, and its radiated field is converted into discrete source histories that are consistent with the prescribed coarse-grid operator. During the online stage, the fine model is no longer solved. The precomputed histories are injected into the global coarse grid for repeated forward simulations and FWI.

This procedure is not an online coupling between fine and coarse grids. One fine-field calculation is required for each physical transmit configuration. The resulting source must be regenerated when the target grid, time step, propagation stencil, source-region medium, or spatial cutoff is changed.

### 2.2. Fine Physical Model

The local fine model is governed by the variable-density scalar acoustic equation

(1) $$\frac{1}{K(x)}\frac{\partial^{2}p_{f}}{\partial t^{2}}-\nabla \;\cdot \;\left[\frac{1}{\rho (x)}\nabla p_{f}\right]=s_{f}\left(x,t\right),$$

where $p_{f}$ is acoustic pressure, *K* is bulk modulus, ρ is density, and $s_{f}$ denotes the physical excitation. The transducer aperture $\Gamma_{A}$ is driven by the prescribed normal velocity $v_{n}\left(t\right)$, equivalently imposing

(2) $${\left.\frac{1}{\rho }\frac{\partial p_{f}}{\partial n}\right|}_{\Gamma_{A}}=-\frac{\partial v_{n}}{\partial t}.$$

The fine model explicitly resolves the physical aperture, acoustic lens, and surrounding water. A smoothly graded absorbing layer is placed at the outer boundary of the fine domain to prevent truncation reflections from entering the retained field. This absorbing layer is independent of the source box defined below.

The source-box transition layer and its complete target-stencil footprint lie in homogeneous water, outside the aperture, lens, and fine-domain absorbing layer. In this region, $\rho =\rho_{0}$ and $K=K_{0}=\rho_{0}c_{0}^{2}$, so Equation (1) reduces to the same source-free constant-density pressure equation used locally on the coarse grid. Density and bulk-modulus contrasts inside the device affect the transferred pressure field but are not averaged into the coarse-grid coefficients. The fine-model excitation and absorbing boundary are not copied to the coarse grid; their role is to produce the outgoing reference field. Differences between the two discrete propagation operators remain in $D_{h}{\left[U_{h}\right]}^{n}$; approximate exterior-field reproduction requires the consistency condition in Equation (15).

The fine simulation produces the reference field $p_{f}\left(x,t\right)$. Only the region required by the source box, its transition layer, and the target finite-difference stencil is transferred to the target grid:

(3) $$U_{h}^{n}=R_{h}p_{f}\left(x,t_{n}\right),$$

where $R_{h}$ is the fine-to-coarse sampling operator. The grids are nested in this study, so $R_{h}$ directly extracts coincident spatial nodes and target time levels without spatial averaging.

### 2.3. Target-Grid Operator

The global coarse-grid field is advanced with the constant-density pressure equation. For a diagonal damping field Σ, the discrete update is

(4) $$\left(I+\Delta t\Sigma \right)p^{n+1}=2p^{n}-\left(I-\Delta t\Sigma \right)p^{n-1}+\Delta t^{2}c^{2}\left(x\right)L_{h}p^{n}+q^{n},$$

where $L_{h}$ is the prescribed target-grid finite-difference operator, Δt is the target time step, c(x) is the sound-speed model, and $q^{n}$ is the discrete source term.

The source box is placed in homogeneous, undamped water. Within this local region, the target operator can therefore be written as

(5) $$D_{h}{\left[u\right]}^{n}=u^{n+1}-2u^{n}+u^{n-1}-\Delta t^{2}c_{0}^{2}L_{h}u^{n},$$

where $c_{0}$ is the water sound speed in the source region. All three source representations use the same physical excitation, aperture center and orientation, propagation grid, time step, medium, boundaries, receiver locations, interpolation rule, and evaluation window. Only the construction of $q^{n}$ is changed.

### 2.4. Three Source Representations

Let *A* be the physical aperture area, $\Delta V_{h}$ the target-cell volume, and $K_{0}=\rho_{0}c_{0}^{2}$ the water bulk modulus. The aperture-integrated excitation represented on the target grid is

(6) $$f_{h}^{n}=\frac{\Delta t^{2}K_{0}A}{\Delta V_{h}}\frac{dv_{n}\left(t_{n}\right)}{dt}.$$

For spatially uniform normal velocity, the volume-injection rate is $Q\left(t\right)=Av_{n}\left(t\right)$, with positive $v_{n}$ defined as injection into water. Its pressure-equation source is $K_{0}\dot{Q}\left(t\right)\delta \left(x-x_{s}\right)$. Replacing the spatial delta by $1/\Delta V_{h}$ and multiplying by $\Delta t^{2}$ in the second-order time update gives Equation (6). Thus, *A* integrates the aperture excitation, $1/\Delta V_{h}$ supplies the discrete source density, and $\Delta t^{2}$ converts pressure acceleration to the pressure-valued increment $f_{h}^{n}$. The units are $s^{2}\;\mathrm{Pa}\;m^{2}\;m^{-3}\;m\;s^{-2}=\mathrm{Pa}$, consistent with $q^{n}$ in Equation (4).

**Figure 1:**
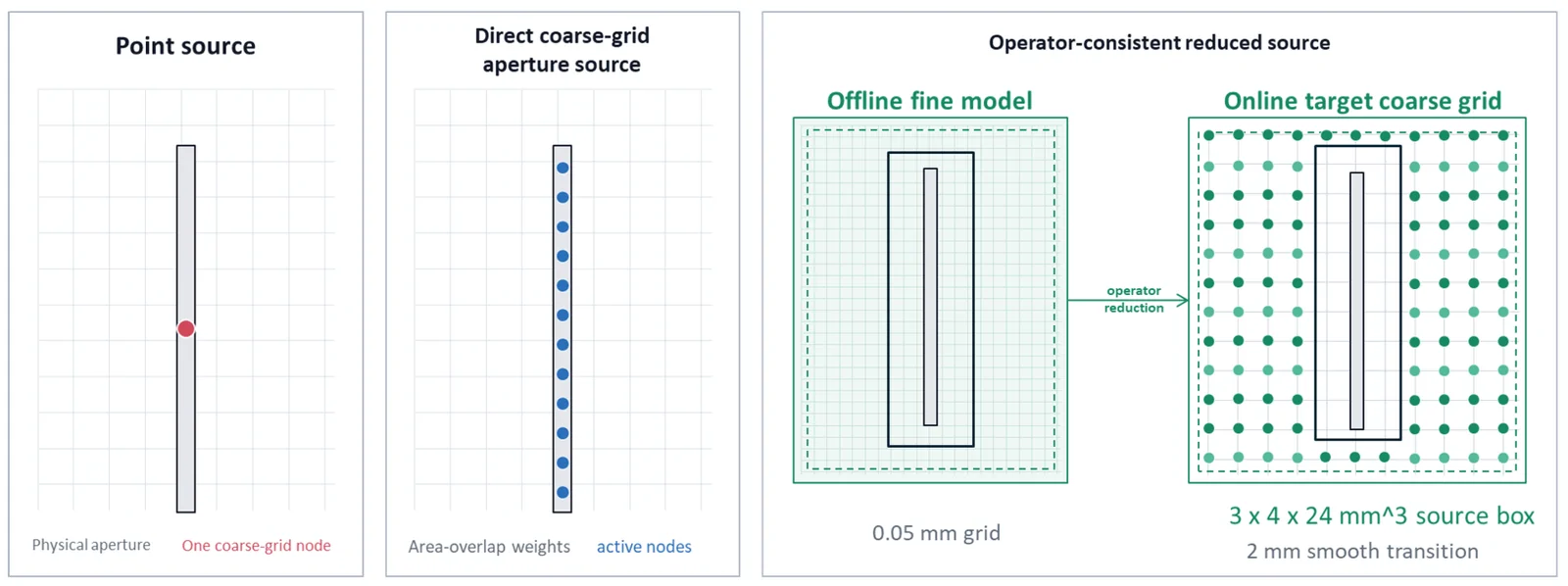
Three source representations on the same target coarse grid. The Point source injects the aperture-integrated excitation at the nearest coarse-grid node. The Direct coarse-grid aperture source conservatively projects the physical aperture onto intersected target cells and applies one shared waveform with overlap weights. The Operator-consistent reduced source uses one offline fine physical simulation and converts the retained exterior field into independent nodal forcing histories supported near the source-box transition layer.

The three representations differ only in how this same physical excitation is assigned to target-grid nodes, as summarized in Figure 1.

#### 2.4.1. Point Source

The Point source injects the complete aperture-integrated waveform at the target-grid node nearest the aperture geometric center:

(7) $$q_{P,i}^{n}=f_{h}^{n}\delta_{i,i_{s}},$$

where $i_{s}$ is the selected source node. This representation retains the source center, temporal waveform, and integrated strength, but discards the aperture extent and spatial phase distribution.

#### 2.4.2. Direct Coarse-Grid Aperture Source

The Direct coarse-grid aperture source conservatively projects the physical aperture onto intersected target-grid cells. If $A_{i}$ is the aperture–cell overlap assigned to active node *i*, then

(8) $$w_{i}=\frac{A_{i}}{\sum_{j}A_{j}},\;q_{A,i}^{n}=w_{i}f_{h}^{n}.$$

This representation preserves the integrated strength and the aperture footprint resolvable by the target grid. However, all active nodes share the same temporal waveform, so lens-induced and subgrid phase differences are not retained.

#### 2.4.3. Operator-Consistent Reduced Source

The Operator-consistent reduced source transfers the fine-model exterior field to the prescribed target-grid operator. A source box first encloses the transducer and lens. A time-independent cutoff χ(x) is then defined as

(9) $$\chi \left(x\right)=\left\{\begin{array}{cc} 0, & \mathrm{inside}\;\mathrm{the}\;\mathrm{source}\;\mathrm{box},\\ 0<\chi <1, & \mathrm{within}\;\mathrm{the}\;\mathrm{smooth}\;\mathrm{transition}\;\mathrm{layer},\\ 1, & \mathrm{in}\;\mathrm{the}\;\mathrm{retained}\;\mathrm{exterior}\;\mathrm{region}. \end{array}\right.$$

The source box is neither a physical boundary nor an online fine-grid region. It removes the device interior from the transferred field and localizes the equivalent forcing.

The retained target-grid field is $\chi U_{h}^{n}$. Applying the local target operator gives

(10) $$D_{h}{\left[\chi U_{h}\right]}^{n}=\chi D_{h}{\left[U_{h}\right]}^{n}+\left(D_{h}{\left[\chi U_{h}\right]}^{n}-\chi D_{h}{\left[U_{h}\right]}^{n}\right).$$

The first term is the propagation residual of the sampled fine field under the target operator. The second term is the commutator between spatial truncation and target-grid propagation. We define this localized term as the Operator-consistent reduced source:

(11) $$q_{O}^{n}=D_{h}{\left[\chi U_{h}\right]}^{n}-\chi D_{h}{\left[U_{h}\right]}^{n}.$$

Because χ is independent of time, the temporal-difference terms cancel exactly, yielding

(12) $$q_{O}^{n}=\Delta t^{2}c_{0}^{2}\left[\chi L_{h}\left(U_{h}^{n}\right)-L_{h}\left(\chi U_{h}^{n}\right)\right].$$

Wherever χ is spatially constant, Equation (12) is zero. The nonzero forcing is therefore confined to the smooth transition layer and the neighboring nodes touched by the finite-difference stencil. Each active node carries an independent temporal history $q_{i}\left(t\right)$; these histories are neither sampled pressures nor weighted or delayed copies of one common waveform.

To reduce storage and injection cost, the temporal root-mean-square (RMS) activity of each node is calculated as

(13) $$E_{i}=\sqrt{\frac{1}{N_{t}}\sum_{n=1}^{N_{t}}{\left(q_{i}^{n}\right)}^{2}}.$$

Only nodes satisfying

(14) $$\frac{E_{i}}{\max_{j}E_{j}}\geq \varepsilon$$

are retained. The number of active nodes consequently depends on the target grid, source box, transition layer, finite-difference stencil, and activity threshold; it is not a fixed physical parameter.

### 2.5. Online Propagation and Applicability

After offline construction, only the coordinates of the active coarse-grid nodes and their independent histories $q_{i}\left(t\right)$ are stored. Online simulations start from the standard quiescent coarse-grid state, inject these histories at the corresponding nodes, and advance Equation (4). The fine model, lens, and fine grid do not participate in subsequent forward or adjoint calculations.

If the sampled reference field satisfies the target propagation equation outside the source box,

(15) $$D_{h}{\left[U_{h}\right]}^{n}\approx 0,$$

then Equation (10) gives

(16) $$D_{h}{\left[\chi U_{h}\right]}^{n}\approx q_{O}^{n}.$$

The localized forcing can therefore reproduce the retained exterior field under the prescribed coarse-grid operator.

The Operator-consistent reduced source is tied to the target spatial operator, time step, source-region medium, source box, and cutoff used during construction. A change in any of these settings requires regeneration of the nodal histories. The method corrects the discrete representation of an unresolved finite-aperture source; it does not compensate for global numerical dispersion. The target grid must therefore already resolve wave propagation over the frequency band of interest.

### 2.6. Comparison Controls and Evaluation Metrics

Numerical and controlled FWI comparisons used no method-specific gain, time shift, or receiver normalization. Hydrophone fields were independently peak-normalized within each measurement plane because that experiment evaluated spatial field shape rather than absolute pressure. In the ring-water experiment, each method was allowed one global scale and one set of per-transmit strength and pointing corrections fitted on water1; these values were frozen before water2 and water3 were evaluated. No per-receiver gain, per-trace time shift, or channel normalization was used for reported ring-water metrics. In real-phantom FWI, the water-derived waveform, water sound speed, and transmit corrections were retained, but one global RMS scale was estimated separately for each source representation from the selected phantom observations under the homogeneous initial model and then frozen for inversion. The real-phantom module therefore assessed complete reconstruction pipelines rather than blind transfer of every amplitude parameter from water.

Field error was quantified by

(17) $$E_{2}=\frac{∥u-u_{\mathrm{ref}}{∥}_{2}}{∥u_{\mathrm{ref}}{∥}_{2}},$$

and spatial or waveform similarity by the normalized cross-correlation

(18) $$\mathrm{NCC}\left(u,v\right)=\frac{\langle u,v\rangle }{{∥u∥}_{2}{∥v∥}_{2}}.$$

Complementary outcomes included median trace error, signed arrival-time residual, 95th-percentile arrival-time error, angular main-lobe axis and width errors, model RMSE, structural-edge cosine similarity, and cross-acquisition repeatability. The exact metric definitions and aggregation rules are provided in Supplementary Methods S2.

## 3. Experimental Design

Five experimental modules were used. The numerical experiment tested source transfer against a converged physical reference; two physical-transducer experiments compared predicted and measured fields and tested blind calibration transfer; controlled FWI tested reconstruction with known truth; and real-phantom FWI assessed imaging-scale feasibility. Table 1 summarizes the purpose, setup, metrics, and supported claim of each module.

### 3.1. Three-Dimensional Numerical Source Experiment

**Figure 2:**
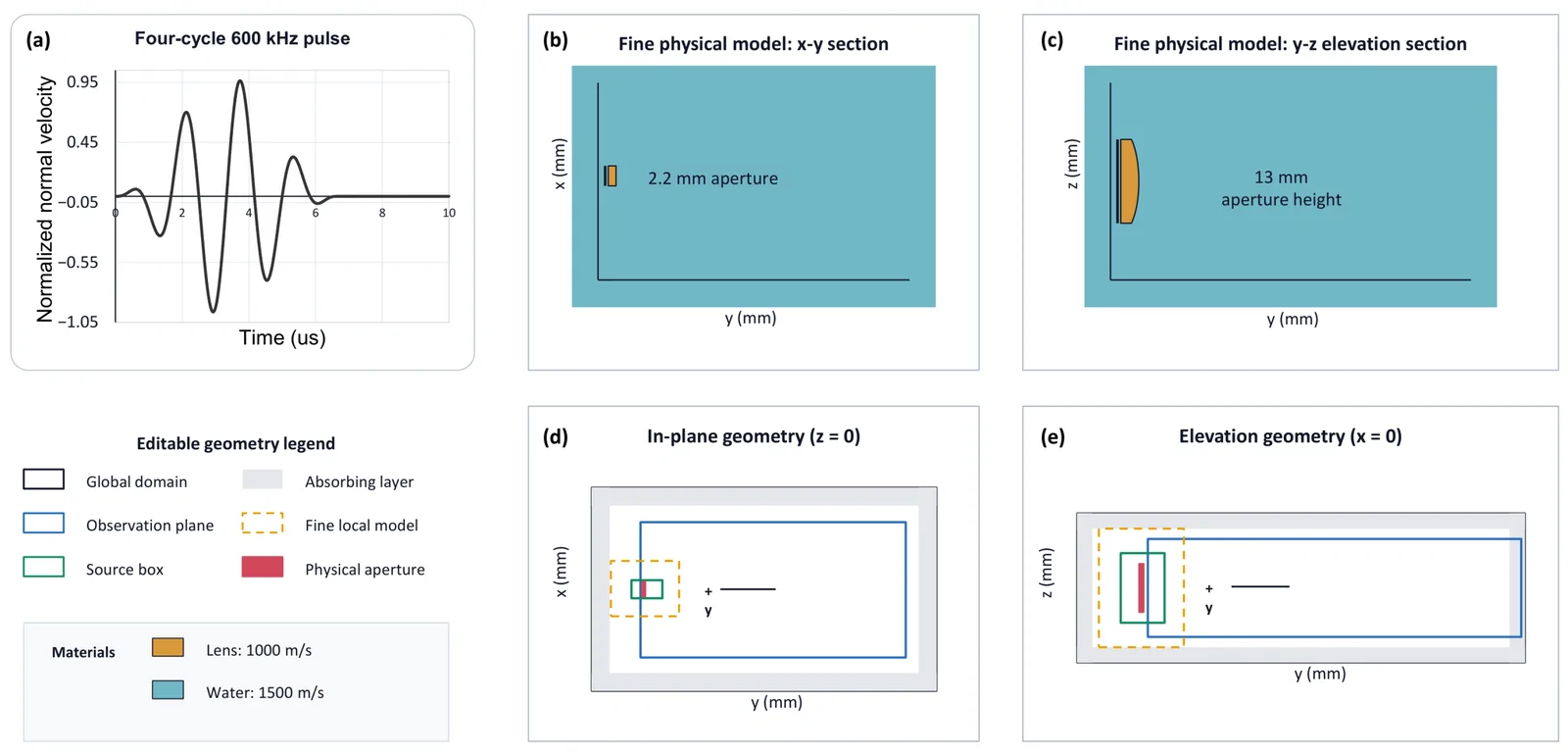
Common three-dimensional numerical configuration. (**a**) Four-cycle, 600 kHz Hann-windowed excitation. (**b**,**c**) Fine physical transducer and lens sections in the in-plane and elevation directions. (**d**,**e**) Fine local model, source box, global propagation domain, orthogonal observation planes, and absorbing layer.

The numerical experiment was designed around the bandwidth and geometry used in the physical study (Figure 2). The medium was lossless water with c=1500 m/s and ρ=1000 kg/m^3^. The lens had c=1000 m/s, ρ=1200 kg/m^3^, and a 20.6 mm elevation-curvature radius. A 2.2×13 mm^2^ uniformly vibrating aperture emitted a four-cycle, 600 kHz Hann-windowed normal-velocity pulse with a peak velocity of 1 m/s. Analysis was restricted to 450–770 kHz.

The fine physical model used spacings of 0.01 mm in-plane and 0.05 mm along the propagation and elevation directions. The target grids used isotropic spacings of 0.25, 0.50, and 0.75 mm. The global domain measured 100×180×60 mm^3^ and was propagated for 180 μs, with fields sampled at 10 MHz. The primary reduced-source inner box measured 3×4×24 mm^3^, followed by a 2 mm quintic smooth transition. Orthogonal in-plane and elevation observation planes were sampled every 0.5 mm. The Courant number was fixed at 0.1 for all fine and coarse calculations. Source-box size, active-support threshold, and target-grid spacing were changed only in their prespecified sensitivity experiments.

### 3.2. Physical Transducer Experiments

#### 3.2.1. Hydrophone Field Measurement

Hydrophone measurements were used as independent field-shape evidence, not to fit the numerical source. The measured in-plane field comprised 41×41 points and the elevation field 31×41 points. Both measured and numerical fields were filtered to 450–770 kHz, converted to RMS pressure, interpolated to the native hydrophone coordinates, and independently normalized by their plane maximum. A single preregistered axial-coordinate correction, $y_{\mathrm{physical}}=y_{\mathrm{reported}}+10$ mm, was applied to every field. No method-specific shift, rotation, spatial scaling, gain fitting, or lens retuning was permitted. The measured water sound speed was 1486.21 m/s; the numerical reference retained the frozen 1500 m/s setting. Thus, agreement with the fine physical reference measured reduction fidelity, whereas agreement with the hydrophone field measured the adequacy of the complete physical model.

#### 3.2.2. Ring-Water Calibration and Blind Validation

The experimental ring comprised eight modules of 256 independently controlled elements, for a total of 2048 elements and a fixed radius of 110 mm. The element height was 13 mm and a front acoustic lens provided broad in-plane emission and elevation confinement. Each acquisition contained 128 sequential transmit events from elements 1:16:2033, 2048 receive channels. Three pure-water acquisitions, water1–water3, were recorded under unchanged hardware settings.

Water1 served as the only calibration acquisition. The water sound speed was 1523.11 m/s. The common waveform was cropped once from the unfiltered opposite-channel signal of shot 33 and was subsequently filtered to 450–770 kHz. Direct-wave strength was defined as RMS amplitude in one fixed 332-sample window. A common 3.8 mm effective in-plane aperture was selected on water1 and then used by both finite-aperture source representations. This fitted width describes the effective response in the analysis band and was not interpreted as a manufactured element dimension. For each transmitter, one relative transmit strength and one pointing offset within ±10 degrees were fitted on water1 from the measured main-lobe profile. Receive directivity was kept flat, and no module-level hierarchy, adjacent-transmitter smoothing, per-receiver gain, or trace-wise time shift was introduced. The complete parameter set was frozen before water2 and water3 were evaluated.

Blind performance was evaluated within ±30 degrees of the water1-derived beam axis. Absolute-amplitude agreement was quantified by sector relative $L_{2}$ error. Directional shape was quantified by per-transmit relative $L_{2}$ error after the measured and predicted profiles were independently normalized by their peak amplitudes; medians were then reported across transmitters. Full-sector maps and representative waveforms were retained as complementary evidence. Table 2 lists the frozen acquisition and reconstruction settings.

### 3.3. Controlled FWI

Controlled FWI tested whether source-field error affected reconstruction when the remaining physics were fixed. A two-dimensional 250 mm square domain contained a 1540 m/s background and three inclusions. The upper-left 1570 m/s ellipse was centered at (−25.0,13.0) mm with semi-axes of 18.5 and 13.0 mm; the right 1518 m/s disk was centered at (23.9,−8.7) mm with a 14.1 mm radius; and the lower 1560 m/s ellipse was centered at (−2.2,−27.2) mm with semi-axes of 11.9 and 17.4 mm. Density was fixed at 1000 kg/m^3^. The phantom was surrounded by a transmitter–receiver ring of radius 110 mm and a 10 mm damping layer.

Independent observations were generated on a 0.25 mm grid, whereas all inversions used a 0.50 mm grid. Thirty-two transmitters and 128 receiver positions were used; for each shot, 65 receivers spanning 90–270 degrees relative to the transmitter were retained. The three methods shared the same four-cycle 600 kHz excitation, initial model of 1540 m/s, 1450–1620 m/s bounds, circular update region, two-cell Gaussian gradient smoothing, and projected line search. The normalized least-squares data objective was evaluated with the same receiver interpolation and time window. Each gradient was normalized by its maximum absolute value inside the update region and proposed a maximum 12 m/s update; trial fractions were 1, 1/2, 1/4, 1/8, 1/16, and 1/32. No method-specific gain, time shift, or channel normalization was applied. A method stopped if no tested fraction decreased the objective or after three consecutive iterations with an RMS model change no greater than 0.10 m/s and a relative loss decrease no greater than 0.5%, subject to a 40-iteration limit. This common rule produced 40 accepted iterations for the Point source, 22 for the Direct coarse-grid aperture source, and 19 for the Operator-consistent reduced source. True-model errors were used only for post hoc evaluation, not for stopping.

The discrete adjoint included damping, the transpose spatial stencil, source injection, and receiver interpolation. Before inversion, a directional derivative test compared the adjoint gradient with centered finite differences and achieved a minimum relative error of $1.98\times 10^{-4}$.

### 3.4. Real-Phantom FWI

Three repeated tissue-mimicking phantom data sets were reconstructed. All measured traces were filtered to 450–770 kHz, padded according to the 262-sample hardware delay, and muted before the geometric source-onset arrival. Each source family retained the waveform, water sound speed, and applicable per-transmit calibration parameters from its water-calibrated parameter package. To account for the remaining method-dependent scale conversion between each exported source package and the differentiable solver, one scalar amplitude factor was estimated for each source representation and phantom acquisition under the homogeneous initial model,

(19) $$\alpha ={\left[\frac{\sum w^{2}d_{\mathrm{obs}}^{2}}{\sum w^{2}d_{\mathrm{syn}}^{2}}\right]}^{1/2},$$

where *w* denotes the fixed preprocessing and receiver weights. The resulting scalar was applied to every shot and receiver and was frozen throughout inversion. No receiver-wise gain or trace-wise time shift was fitted. Consequently, the real experiment compared three complete reconstruction pipelines, each using one source representation; it was not a source-only ablation or a blind test of water-to-phantom amplitude transfer. For every shot, the opposite 513 receivers (the geometric opposite element ±256 channels) were used.

All reconstructions used a 555×555 grid with 0.45 mm spacing, a water initial model, 1450–1600 m/s bounds, 128 transmissions, and 200 prespecified iterations. The scalar acoustic solver used an eighth-order spatial discretization, a 10 mm damping layer, and automatic differentiation. Adam optimization used an initial learning rate of 1.0, a factor of 0.90 every 10 iterations, four-shot batches, a temporal residual stride of four, Gaussian gradient smoothing with a two-cell standard deviation, and a maximum per-iteration update of 2 m/s. These settings provide a shared computational budget and constrained update rule, not a method-specific optimum. The batch size and temporal stride limit memory use and residual-evaluation cost; learning-rate decay reduces late-stage step sizes, while gradient smoothing and the update cap limit spatially oscillatory or excessively large changes. The same settings were used for all methods to avoid confounding source representation with optimizer tuning. No exhaustive hyperparameter search was performed, so these values are not claimed to be optimal. Checkpoints were saved at iterations 80, 120, 160, and 200. Iteration 200 was reported for all methods; checkpoint histories were used to assess late evolution, not to select a method-specific optimum.

After reconstruction, a structural section was rigidly registered to each acquisition using the phantom-cylinder geometry. The registration and interior mask were shared by all methods within that acquisition. The structural images were used only for post hoc edge evaluation and were not inputs to inversion, source calibration, stopping, or parameter selection. Reported outcomes were terminal training loss, structural-edge cosine similarity, mean reconstruction-edge distance, RMS model change between iterations 160 and 200, interior sound-speed distribution, and pairwise cross-acquisition update NCC. The acquisition, not an individual image pixel or receiver trace, is the unit of replication. Means summarize performance across the three repeated acquisitions, and error bars show population standard deviation, calculated with a denominator of three, for this archived set. They describe the magnitude and consistency of the observed differences under repeated acquisition of the same phantom. They do not estimate confidence intervals or establish population-level superiority: three acquisitions of one phantom do not sample variability across independent phantoms, tissue types, or patients. No significance tests are inferred from error-bar overlap.

## 4. Results

### 4.1. Numerical Reproduction of the Fine Exterior Field

**Figure 3:**
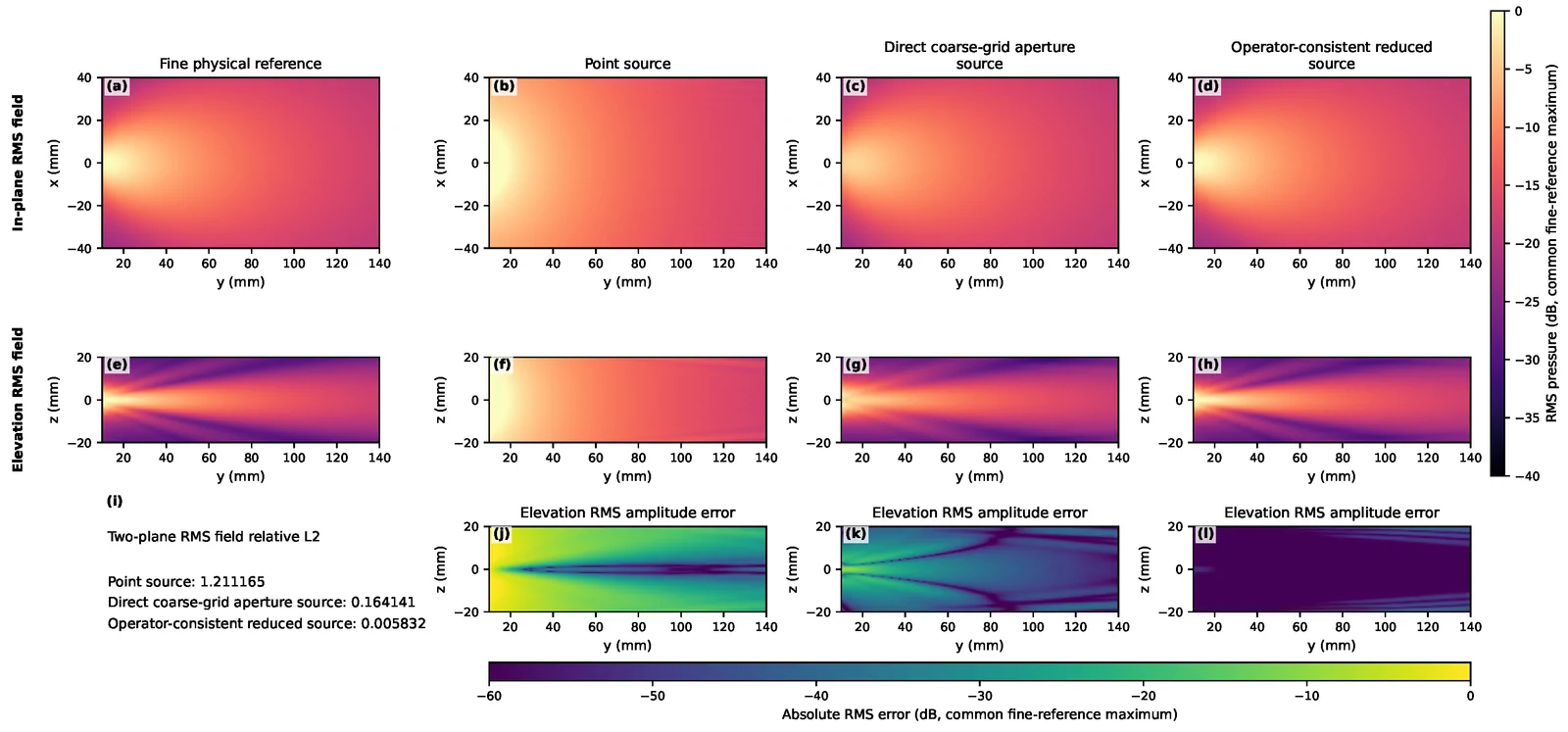
Orthogonal RMS fields and errors on the 0.50 mm target grid. Panels (**a**–**d**) show the in-plane RMS pressure fields, and panels (**e**–**h**) show the elevation fields, for the fine physical reference, Point source, Direct coarse-grid aperture source, and Operator-consistent reduced source, in that order. All fields use the common fine physical reference maximum and the same [−40,0] dB scale. Panel (**i**) reports the two-plane RMS-field relative $L_{2}$ errors. Panels (**j**–**l**) show elevation-plane absolute errors for the Point source, Direct coarse-grid aperture source, and Operator-consistent reduced source, respectively, on a common [−60,0] dB scale.

Figure 3 compares the orthogonal RMS fields and their errors. On the 0.50 mm grid, two-plane RMS-field relative $L_{2}$ errors were 1.2112 for the Point source, 0.1641 for the Direct coarse-grid aperture source, and 0.00583 for the Operator-consistent reduced source. The Operator-consistent reduced source reduced this error by 99.5% relative to the Point source and by 96.4% relative to the Direct coarse-grid aperture source. Its spatial NCC values were 0.999996 in-plane and 0.999963 in elevation.

**Figure 4:**
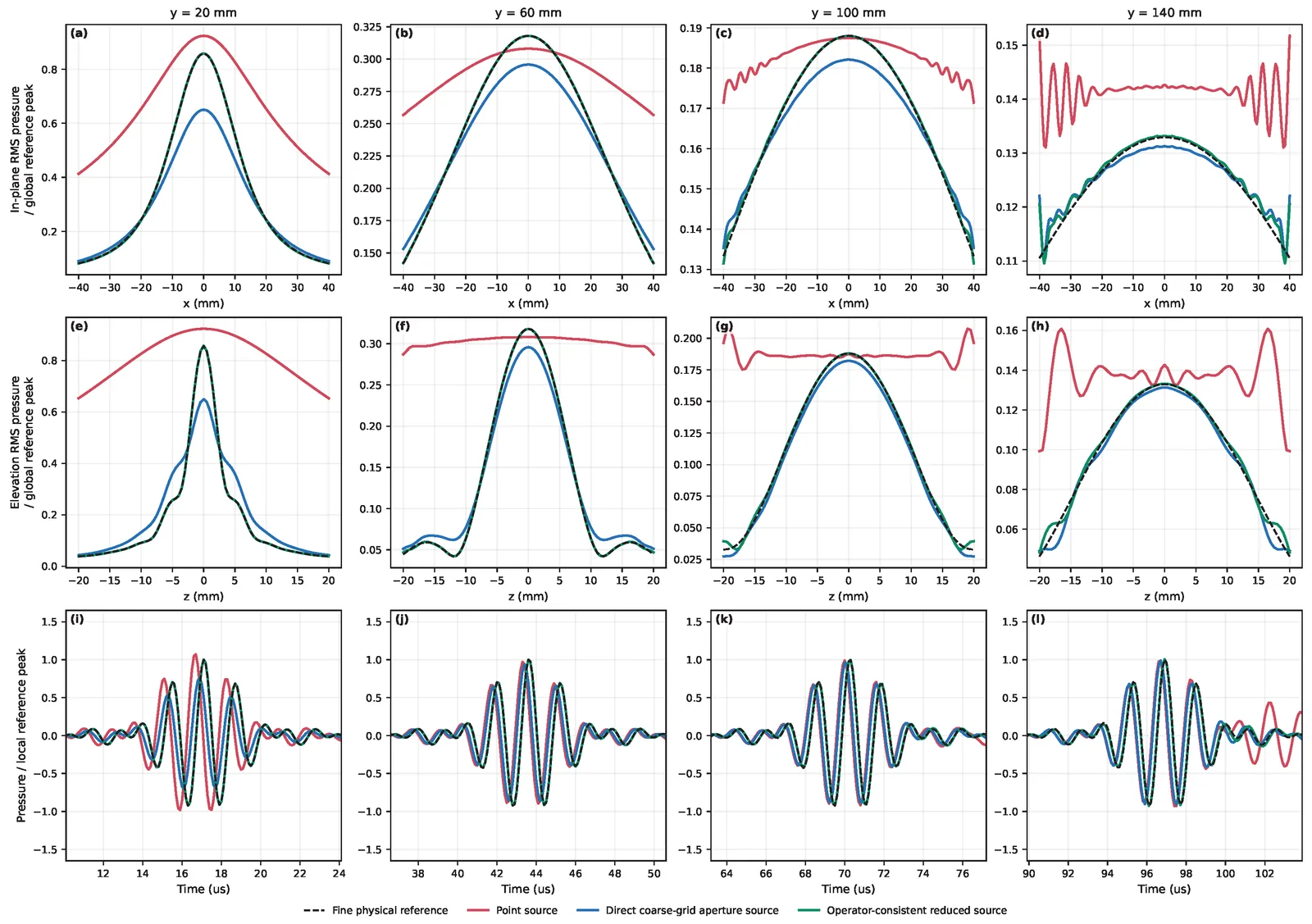
Orthogonal beam profiles and representative received waveforms. Columns correspond to propagation distances of 20, 60, 100, and 140 mm. Panels (**a**–**d**) and (**e**–**h**) show in-plane and elevation RMS pressure profiles normalized by the global fine physical reference maximum. Panels (**i**–**l**) show on-axis waveforms normalized by the local fine physical reference peak, without method-specific gain or time alignment. Black, red, blue, and green curves denote the fine physical reference, Point source, Direct coarse-grid aperture source, and Operator-consistent reduced source, respectively.

**Figure 5:**
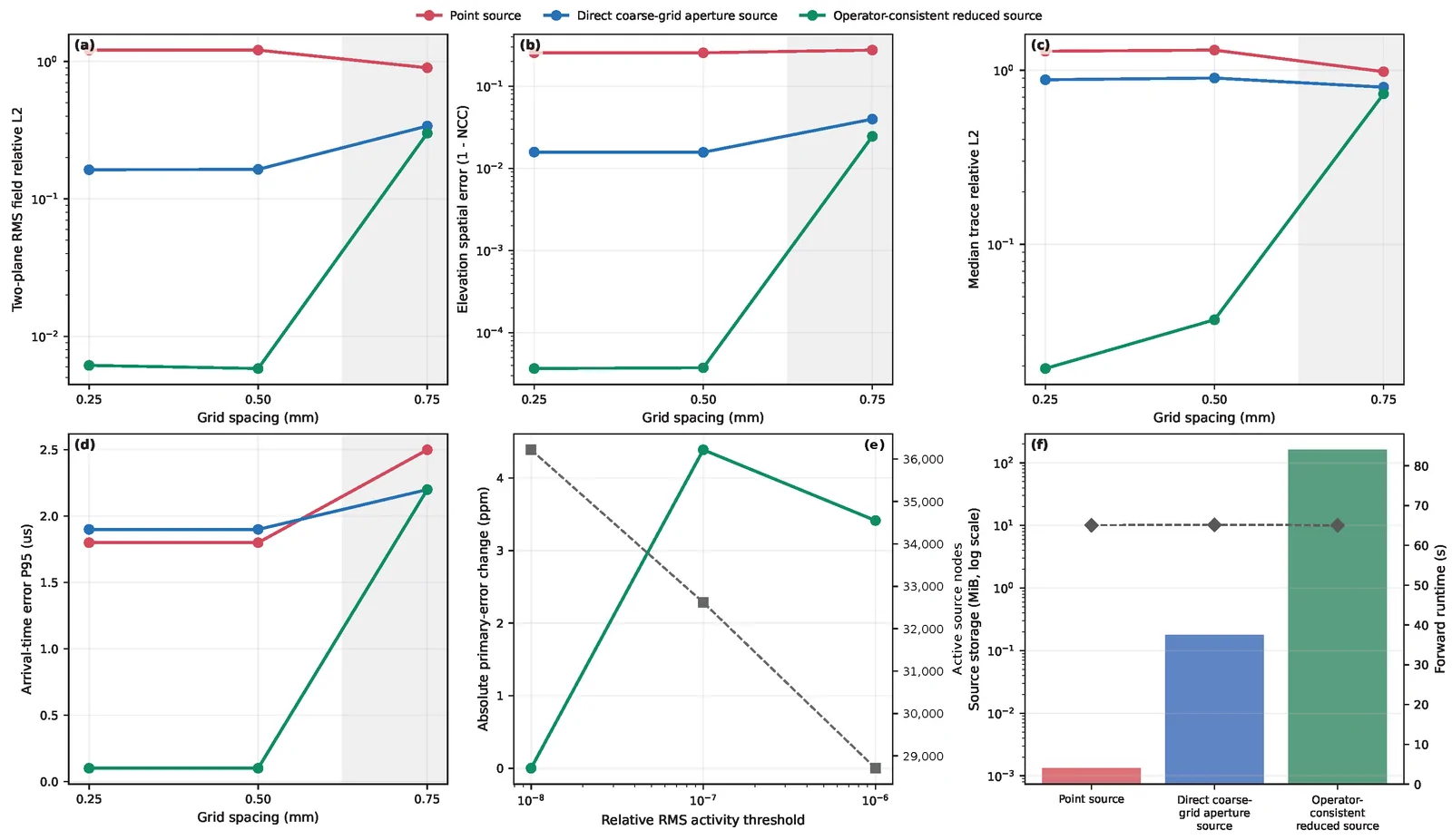
Accuracy, convergence, and computational cost. (**a**) Two-plane RMS-field relative $L_{2}$ error. (**b**) Elevation spatial-shape error, expressed as 1−NCC. (**c**) Median trace relative $L_{2}$ error. (**d**) 95th-percentile arrival-time error. (**e**) Sensitivity of the Operator-consistent reduced source to the relative RMS activity threshold. (**f**) Source storage and forward runtime on the 0.50 mm target grid. The shaded region marks the 0.75 mm condition. In (**e**), the gray dashed line with square markers gives the active source-node count on the right axis. In (**f**), the black dashed line with diamond markers gives forward runtime on the right axis; the bars show source storage on the left axis.

Figure 4 shows the beam profiles and representative received waveforms; Figure 5 summarizes accuracy, convergence, and computational cost. Representative traces yielded median relative $L_{2}$ errors of 1.3074, 0.9033, and 0.03691 for the Point source, Direct coarse-grid aperture source, and Operator-consistent reduced source, respectively. Median trace NCC values were 0.3494, 0.5854, and 0.9993. The corresponding 95th-percentile arrival-time errors were 1.8–1.9 μs for the baselines and 0.1 μs for the Operator-consistent reduced source. No method-specific gain or time alignment was applied.

For the Operator-consistent reduced source, the two-plane errors at 0.25 and 0.50 mm were 0.00615 and 0.00583. The primary $10^{-7}$ activity threshold retained 32,619 nodes and 161.8 MiB of source histories. Across thresholds from $10^{-8}$ to $10^{-6}$, the two-plane error remained approximately 0.00583, while the active support decreased from 36,219 to 28,709 nodes. Enlarging the source box from 3×4×24 to 7×8×24 mm^3^ changed the primary error by 1.15%.

### 4.2. Physical Transducer Validation

#### 4.2.1. Hydrophone Field Comparison

**Figure 6:**
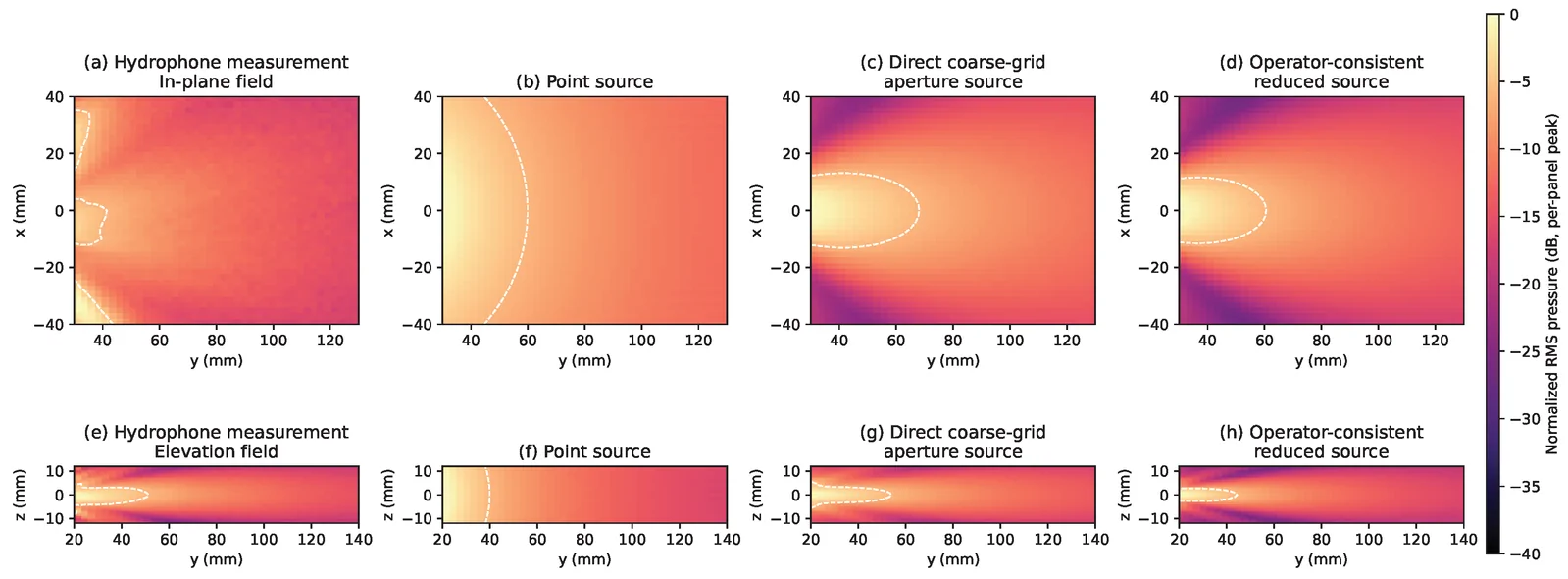
Hydrophone field comparison on two orthogonal planes. Band-limited 450–770 kHz RMS pressure fields measured by the hydrophone and predicted by the three source representations are shown on the native measurement grids. Panels (**a**–**d**) show the in-plane fields, and panels (**e**–**h**) show the elevation fields; within each row, the order is hydrophone measurement, Point source, Direct coarse-grid aperture source, and Operator-consistent reduced source. Each field is independently peak-normalized within its plane and displayed on the same [−40,0] dB scale; white contours mark −6 dB. One preregistered +10 mm axial-coordinate correction is applied to all fields, without method-specific alignment or lens retuning.

**Figure 7:**
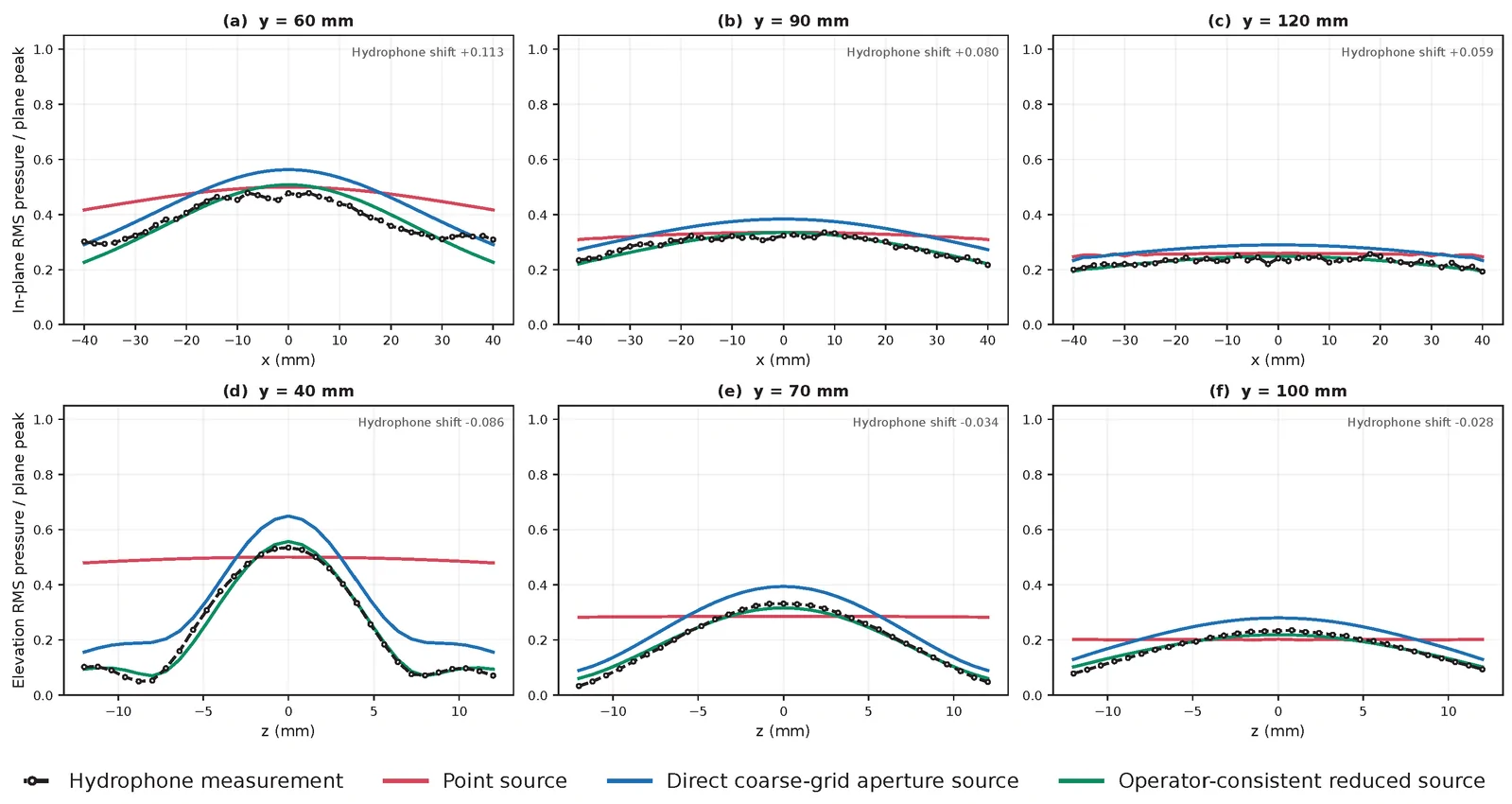
Representative hydrophone and numerical RMS pressure profiles. Panels (**a**–**c**) show in-plane profiles sampled at 60, 90, and 120 mm, respectively; panels (**d**–**f**) show elevation profiles at 40, 70, and 100 mm, respectively. Each field is first normalized by its plane maximum. One explicitly reported additive hydrophone offset is fitted independently in each panel to the Operator-consistent reduced source for profile-shape visualization; the offsets are not used in Figure 6 metrics.

Figure 6 compares the measured and predicted fields, and Figure 7 shows representative profiles. Relative to the frozen fine physical reference, the Operator-consistent reduced source yielded relative $L_{2}$ errors of 0.00240 in-plane and 0.00215 in elevation, with NCC values of 0.999997 and 0.999998. Relative to the hydrophone measurements, its in-plane and elevation relative $L_{2}$ errors were 0.5253 and 0.2438. All measured and predicted fields were evaluated on their native grids with common normalization and display limits.

Profiles sampled at fixed propagation distances quantified the transverse beam shapes. In-plane profiles were evaluated at 60, 90, and 120 mm, and elevation profiles at 40, 70, and 100 mm. The additive measurement offsets shown for profile visualization were excluded from the two-dimensional field metrics.

#### 4.2.2. Blind Ring-Water Transfer

**Figure 8:**
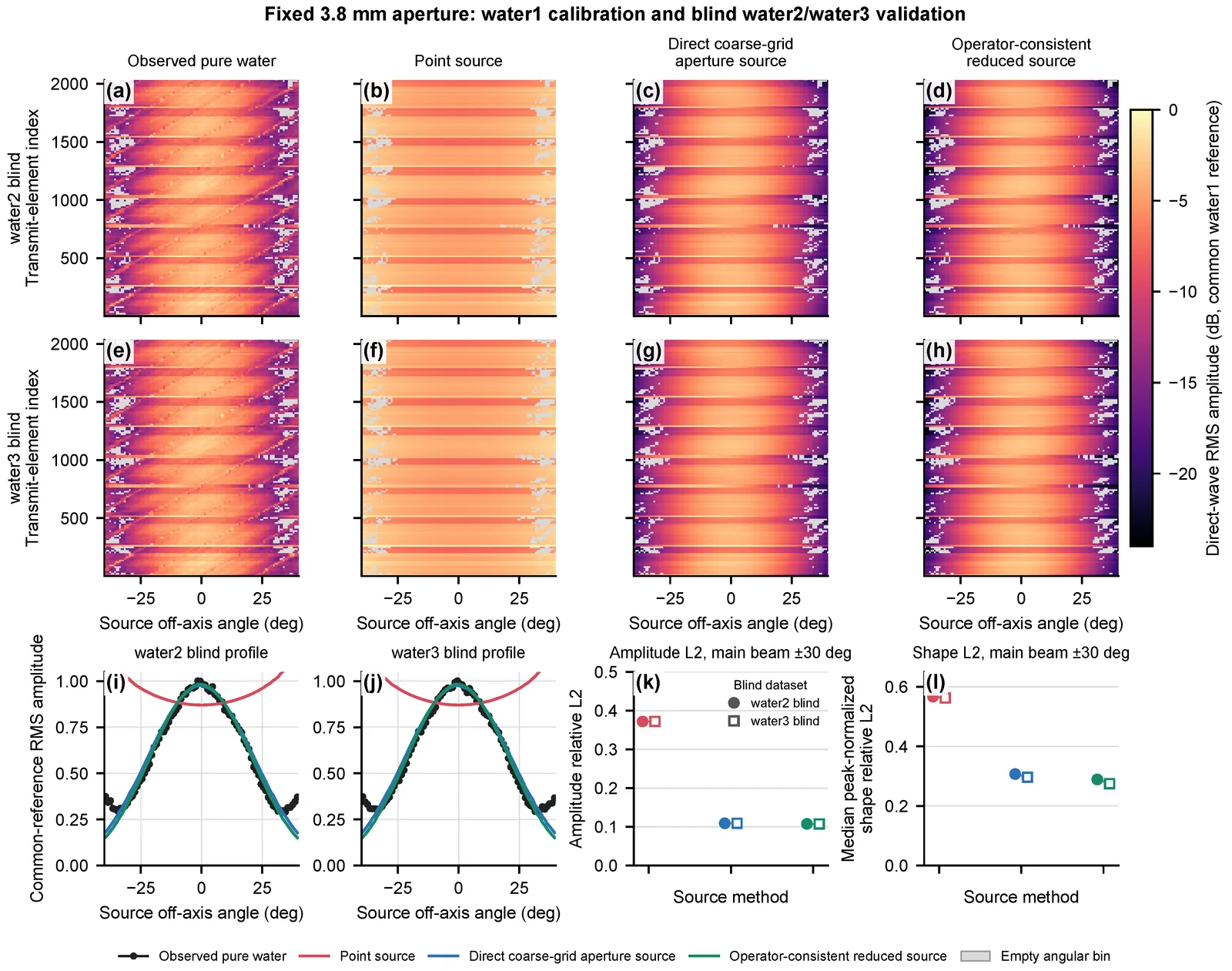
Blind 2048-element ring-water validation with the fixed 3.8 mm effective aperture selected using water1. Per-transmit relative strength and pointing offset were calibrated using water1 only; water2 and water3 were evaluated without refitting. Panels (**a**–**d**) and (**e**–**h**) show water2 and water3 angular-strength maps, respectively; each row shows the observations, Point source, Direct coarse-grid aperture source, and Operator-consistent reduced source, in that order. Panels (**i**,**j**) show the corresponding median blind profiles. Panel (**k**) reports main-beam amplitude relative $L_{2}$ error, and panel (**l**) reports median peak-normalized directional-shape relative $L_{2}$ error. Effective-aperture sensitivity is reported in Figure S12. No per-receiver gain, per-trace time shift, or channel normalization was used.

Figure 8 summarizes blind ring-water validation. The parameters estimated from water1 were frozen before validation on water2 and water3. Within the ±30-degree main-beam sector, water2 absolute-amplitude relative $L_{2}$ errors were 0.3720 for the Point source, 0.1091 for the Direct coarse-grid aperture source, and 0.1075 for the Operator-consistent reduced source. The corresponding water3 values were 0.3722, 0.1091, and 0.1076. Median normalized directional-shape relative $L_{2}$ errors were 0.5671, 0.3071, and 0.2890 for water2 and 0.5625, 0.2965, and 0.2749 for water3.

### 4.3. Controlled FWI

**Figure 9:**
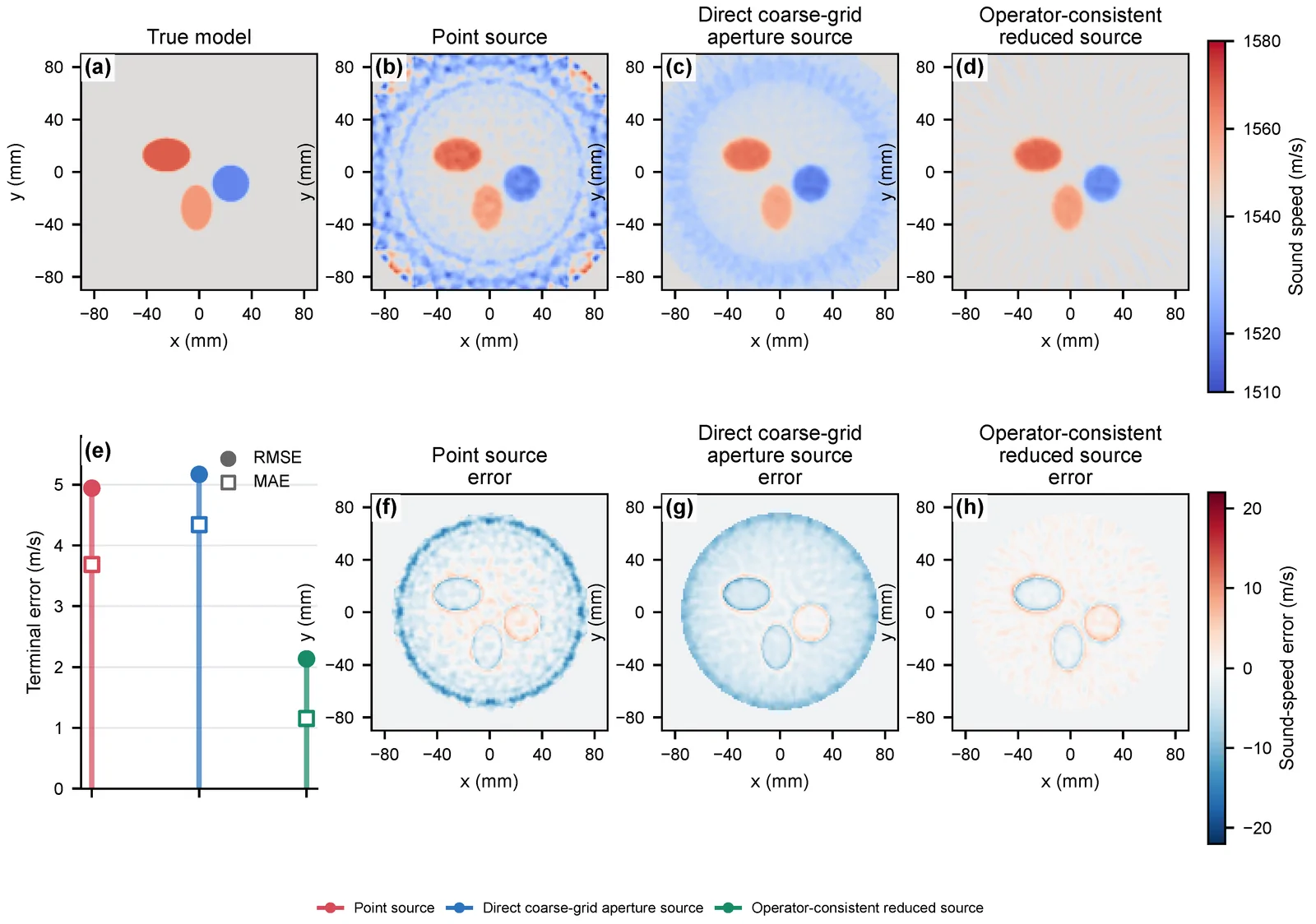
Controlled FWI with independent observations. Panels (**a**–**d**) show the true model and terminal reconstructions for the Point source, Direct coarse-grid aperture source, and Operator-consistent reduced source, in that order, on one shared 1510–1580 m/s scale. Panel (**e**) reports terminal ROI root-mean-square and mean absolute errors. Panels (**f**–**h**) show the corresponding method-specific error maps on a common zero-centered scale. Observations were generated on a 0.25 mm grid, whereas all inversions used the same 0.50 mm grid, 32 transmissions, receiver aperture, initialization, bounds, update mask, and line-search rule.

Figure 9 compares the true model, terminal reconstructions, and reconstruction errors. The initial-model region-of-interest (ROI) RMSE was 8.26 m/s. Terminal RMSE values were 4.94 m/s for the Point source, 5.17 m/s for the Direct coarse-grid aperture source, and 2.14 m/s for the Operator-consistent reduced source. The corresponding model correlations were 0.883, 0.920, and 0.969. All inversions used the same independently generated observations and inversion controls.

### 4.4. Real-Phantom FWI

**Figure 10:**
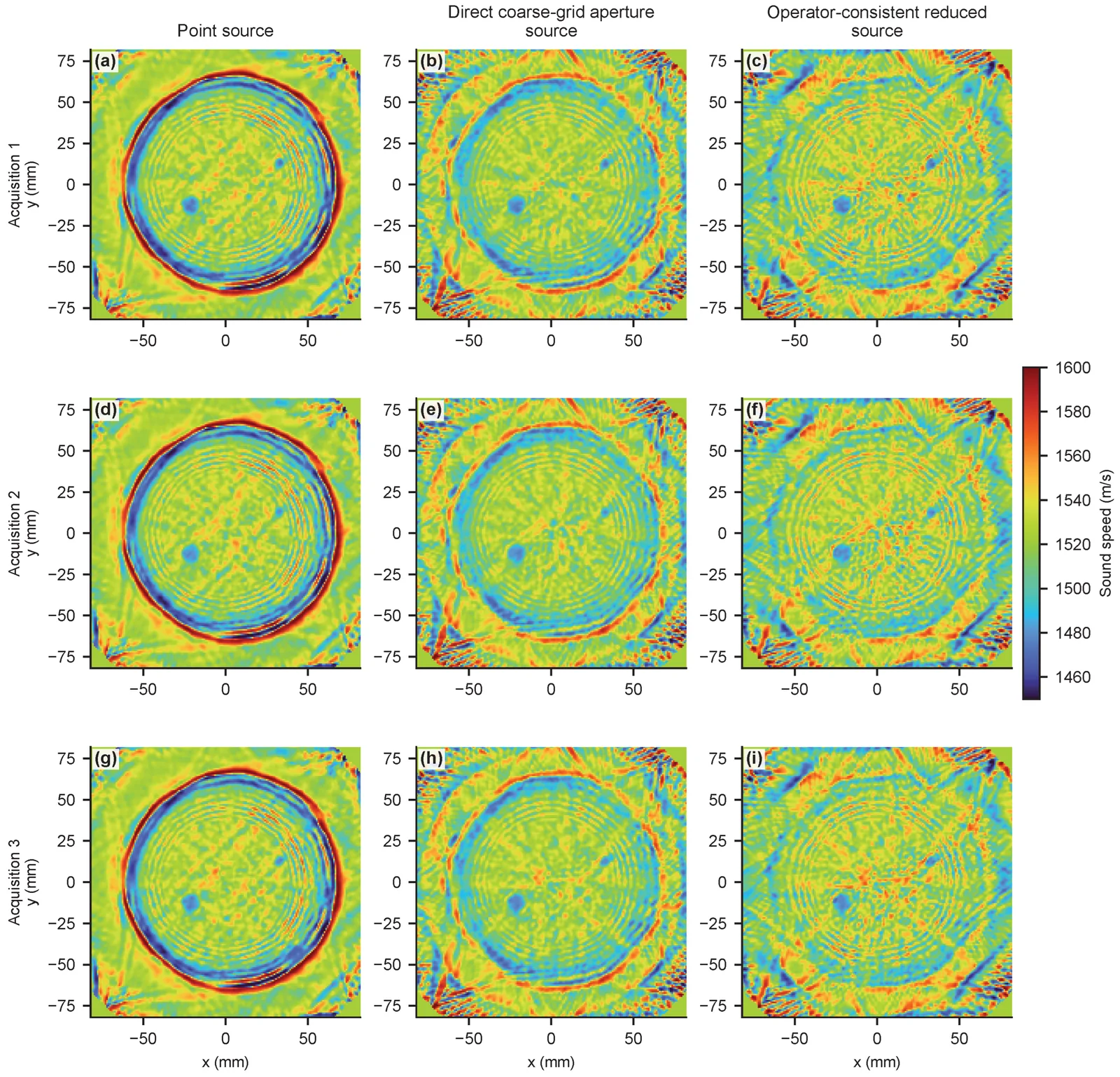
Real-phantom FWI across three repeated acquisitions. Panels (**a**–**c**), (**d**–**f**), and (**g**–**i**) correspond to acquisitions 1, 2, and 3, respectively. Columns denote Point source, Direct coarse-grid aperture source, and Operator-consistent reduced source. All panels show the prespecified iteration-200 models on the same 1450–1600 m/s scale and physical field of view. Structural reference sections were used only for post hoc evaluation.

Figure 10 shows the reconstructions from all three acquisitions. Each source representation recovered a low-speed phantom structure in all three acquisitions. The Operator-consistent reduced source achieved the highest mean structural-edge cosine similarity, 0.567±0.023, compared with 0.547±0.004 for the Direct coarse-grid aperture source and 0.507±0.026 for the Point source. Neither finite-aperture representation reached the prescribed velocity bounds.

**Figure 11:**
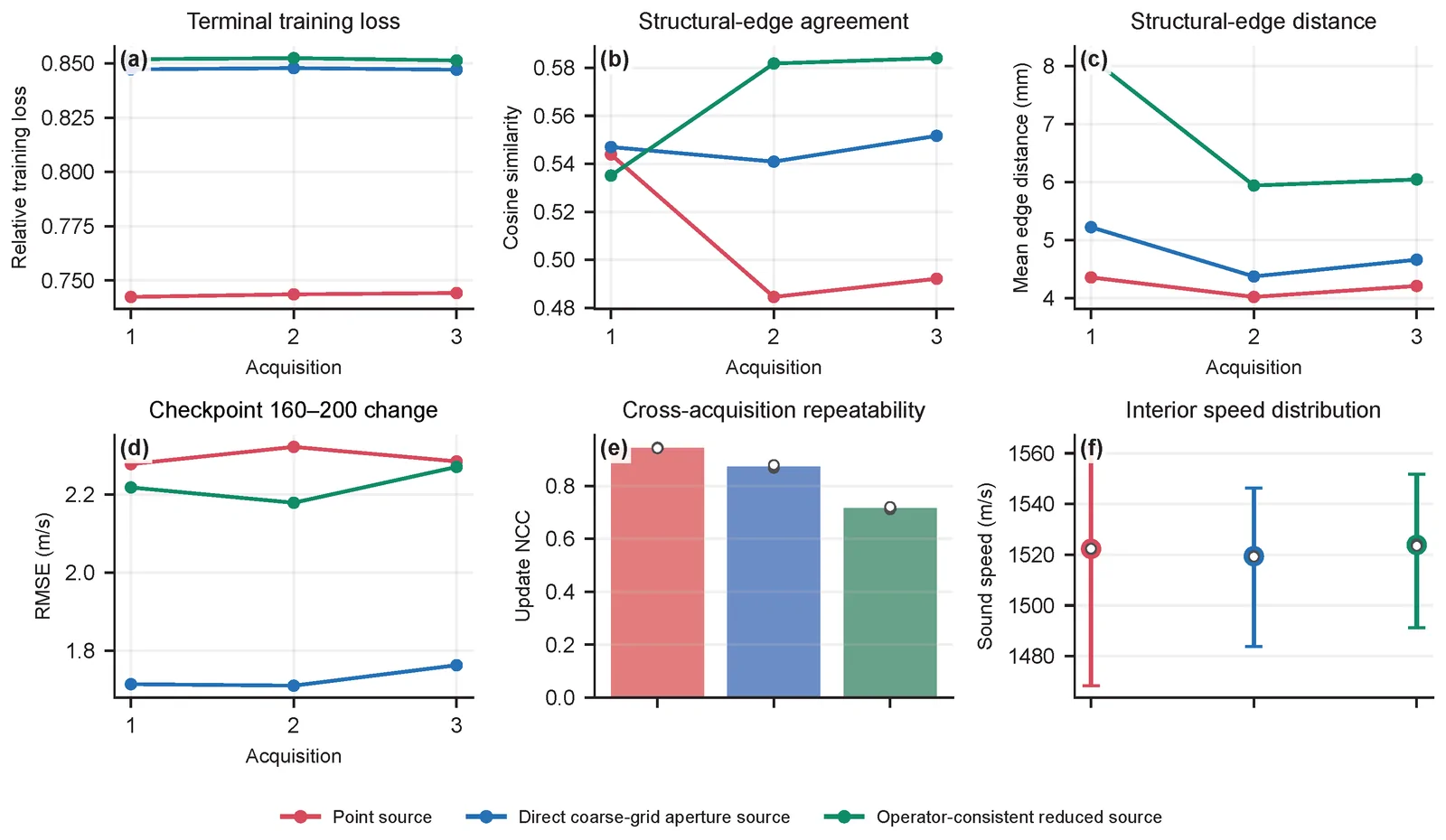
Quantitative assessment of the real-phantom reconstructions. (**a**) Terminal relative training loss. (**b**) Structural-edge cosine similarity. (**c**) Mean reconstruction-edge distance. (**d**) RMS model change between iterations 160 and 200. (**e**) Pairwise cross-acquisition NCC of reconstructed updates. (**f**) Interior sound-speed median and 5th–95th percentile interval. Error bars in (**a**–**d**) denote population standard deviation over the three repeated acquisitions. Training loss is not a held-out validation loss, and structural metrics are post hoc evaluation only.

The prespecified training, structural, convergence, repeatability, and sound-speed statistics are summarized in Figure 11; their cross-metric interpretation is provided in Section 5.

Table 3 summarizes the primary quantitative outcomes.

## 5. Discussion

The numerical results show that finite-aperture source transfer must be matched to the target propagation operator. On the verified 0.50 mm grid, the Operator-consistent reduced source reproduced the fine exterior field with a two-plane relative error of 0.00583 and a median trace NCC of 0.9993. This agreement resulted from independent nodal source histories derived from the action of the target stencil across the cutoff transition, rather than from applying one common waveform to additional nodes.

The grid-spacing sweep also identified the range in which source reduction remained meaningful. At 0.75 mm, field and arrival-time errors increased for all three representations, indicating that propagation error in the target grid had become limiting. This behavior is consistent with the established dependence of finite-difference accuracy on the spatial operator and sampling interval [8,29]. Operator consistency cannot correct an under-resolved propagation stencil; it only constructs the forcing required by the selected stencil [24]. Conversely, when the aperture is adequately resolved and a common waveform describes its active cells, the Direct coarse-grid aperture source is simpler and requires less storage. The Operator-consistent reduced source is most useful when the grid resolves the propagation band but not the device-scale geometry or spatial phase.

The hydrophone experiment separated source-reduction accuracy from the accuracy of the physical transducer model. The Operator-consistent reduced source reproduced the frozen fine-model field almost exactly, whereas the best agreement with measurement depended on the observation plane and metric. The Point source gave the highest in-plane NCC, while the Direct coarse-grid aperture source gave the lowest elevation relative error. Thus, source reduction preserves both the information and the residual error contained in the fine model. This observation agrees with calibrated-transducer studies showing that multilayer electromechanical response, element position and orientation, and elevation focusing can affect measured and predicted fields [12,13,16,20]. The reduction step cannot by itself correct uncertainties in lens geometry, material properties, electromechanical response, or coordinates. The small errors against the frozen fine reference therefore measure numerical transfer fidelity, whereas the larger measurement errors also include physical-model and acquisition mismatch. Measurement noise, hydrophone positioning and calibration uncertainty, and differences between modeled and experimental conditions are additional possible contributors; the present data do not separate their individual contributions.

In the ring-water experiments, the two finite-aperture representations produced nearly identical main-beam amplitude errors after the same 3.8 mm effective aperture was selected using water1. Their remaining difference was smaller than the improvement obtained by correcting the effective aperture width. The small advantage in absolute-amplitude error was consistent across water2 and water3, but its magnitude does not establish a substantial practical advantage over the Direct coarse-grid aperture source in this configuration. These blind tests support transfer of the frozen main-beam calibration, not universal superiority or accurate sidelobe reproduction. This result is consistent with water-calibration studies showing that effective source directivity, rather than nominal element geometry alone, can determine the useful FWI source representation [15]. The fitted width should therefore be interpreted as a band-limited effective parameter, not as a manufactured dimension. Residual disagreement in sidelobes and waveforms may arise from receive directivity, element variation, housing, attenuation, density, or coordinate error. Previous calibration studies likewise identify transducer and coordinate uncertainties as separate sources of model–data mismatch [15,16,20].

The controlled FWI experiment linked source-field error to reconstruction after the other modeled factors were fixed. The Operator-consistent reduced source achieved the lowest model RMSE and the highest model correlation. The two baseline methods were not ranked identically by data loss and model error, which shows that a lower data residual does not necessarily imply a more accurate material estimate when source mismatch is absorbed by model updates. Similar behavior has been reported when improved transducer representations reduced structured artifacts or reconstruction error in FWI [15,16,30].

The real-phantom experiment did not produce the same method ranking for every metric. The Operator-consistent reduced source achieved the highest structural-edge cosine similarity and avoided bound saturation, but it also had the largest mean reconstruction-edge distance and the lowest cross-acquisition update NCC. The Point source had the lowest training loss and the highest repeatability, whereas the Direct coarse-grid aperture source had the smallest RMS model change between iterations 160 and 200. Each source representation used its own frozen global scale, and the model omitted receiver response, housing, attenuation, density variation, and three-dimensional effects. These results therefore compare complete reconstruction pipelines and should not be interpreted as a source-only ranking.

A practical choice among the three representations depends on the required physics and available calibration. The Point source is sufficient when source location and integrated strength are the only required properties; dedicated off-grid or finite-difference-consistent formulations are available when subgrid placement is important [23,24]. The Direct coarse-grid aperture source is suitable when the aperture is adequately resolved and one temporal waveform describes its active cells, as in line-shaped or spatially weighted element models [14]. The Operator-consistent reduced source is appropriate when unresolved geometry or spatial phase affects the exterior field and a reliable fine reference is available, consistent with distributed finite-difference injection principles [27,28]. In every case, the target grid, time step, boundaries, receiver interpolation, and source-region medium should be verified before the source representation is assessed.

The present study used lossless scalar acoustics, fixed or simplified density, two normalized hydrophone planes rather than an absolute-pressure volume, effective ring geometry without receive directivity, and two-dimensional real-phantom FWI without held-out transmitters. These limitations are relevant to full-wave acoustic propagation and calibrated transducer modeling [11,13,20]. Future work should measure receiver responses and incorporate attenuation and density variation to test whether they explain the remaining experimental amplitude and waveform residuals. Three-dimensional forward and adjoint models are needed to assess elevation effects omitted by the present two-dimensional phantom inversions. Transmit events should also be reserved before calibration and optimization, with checkpoint selection based on their validation loss rather than training loss. This would test whether late model changes improve prediction of unseen data or reflect overfitting.

From an end-user perspective, the present results demonstrate technical feasibility, not clinical readiness. The controlled model RMSE, phantom structural agreement, and repeat-acquisition statistics are useful engineering measures, but they were not evaluated against prespecified clinical acceptance criteria. Practical application requires task-specific assessment of sound-speed bias, spatial resolution, contrast recovery, repeatability across subjects and systems, and total reconstruction time. The reported source-reduction accuracy and reuse efficiency alone do not establish diagnostic performance or a clinically acceptable workflow.

## 6. Conclusions

This study formulated the transfer of a device-resolved finite-aperture field to a prescribed coarse-grid FDTD solver as an operator-specific forcing problem. The resulting Operator-consistent reduced source converts one offline fine-grid calculation into reusable source histories localized near the source-box transition layer. On a target grid that resolved the propagation band, it reproduced the fine exterior field with lower spatial, waveform, and arrival-time errors than the Point source and Direct coarse-grid aperture source. Hydrophone and blind ring-water measurements provided independent physical comparisons, while controlled and real-phantom FWI evaluated its use in inversion. The method provides a reusable way to retain unresolved finite-aperture radiation features in imaging-scale finite-difference calculations without coupling the fine and coarse models during repeated simulations.

## Figures and Tables

**Table 1:** Purpose, setup, metrics, and supported claim for each experimental module.

Module	Purpose	Primary Setup	Metrics	Supported Claim
Three-dimensional numerical experiment	Isolate source transfer	Three methods against one converged fine physical reference	Field and trace errors, NCC, arrival error	Exterior-field reproduction on a verified target operator
Hydrophone experiment	Compare predicted and measured field shapes	Two orthogonal measured planes and frozen simulations	Plane relative $L_{2}$, NCC, profiles	Retention of the fine model and measured-field agreement
Ring-water experiment	Evaluate calibration transfer	water1 calibration; blind water2/water3 validation	Main-beam amplitude and normalized shape errors	Transfer across repeated full-array acquisitions
Controlled FWI	Test reconstruction with known truth	Independent observations and common inversions	Model RMSE and correlation	Reconstruction effect under fixed non-source controls
Real-phantom FWI	Assess imaging-scale feasibility	Three repeated phantom acquisitions	Training, structural, convergence, and repeatability metrics	Feasibility in a complete experimental pipeline

**Table 2:** Frozen parameters for ring-water validation and real-phantom FWI.

Parameter	Value
Array architecture	2048 elements in eight 256-element modules; 110 mm radius
Transmit sequence	128 transmitters, elements 1:16:2033
Analysis band	450–770 kHz
Calibration split	water1 calibration; water2 and water3 blind validation
Water sound speed	1523.11 m/s from water1
Effective in-plane aperture	3.8 mm selected on water1 and shared by both finite-aperture methods
Transmit corrections	Per-transmit strength and pointing offset within ±10 degrees; no receiver-specific gain or trace-wise time shift
Blind evaluation sector	±30 degrees around the water1-derived main-beam axis
FWI receiver sector	Opposite element ±256 channels (513 receivers per shot)
FWI grid and bounds	555×555, 0.45 mm; 1450–1600 m/s
FWI schedule	128 shots, 200 iterations; checkpoints at 80, 120, 160, and 200

**Table 3:** Primary quantitative outcomes. Lower is better for relative $L_{2}$ and RMSE; higher is better for NCC, correlation, and structural-edge cosine similarity.

Experiment	Metric	Point Source	Direct Coarse-Grid Aperture Source	Operator-Consistent Reduced Source
Numerical, 0.50 mm	Two-plane RMS-field relative $L_{2}$ error	1.2112	0.1641	0.00583
Numerical, 0.50 mm	Median trace NCC	0.3494	0.5854	0.9993
Blind ring-water, water2	Main-beam amplitude relative $L_{2}$	0.3720	0.1091	0.1075
Blind ring-water, water3	Main-beam amplitude relative $L_{2}$	0.3722	0.1091	0.1076
Controlled FWI	Model RMSE (m/s)	4.94	5.17	2.14
Controlled FWI	Model correlation	0.883	0.920	0.969
Real-phantom FWI	Mean structural-edge cosine similarity	0.5069	0.5465	0.5670

## Data Availability

Code, versioned configurations, and machine-readable result tables will be deposited in a public repository. Availability of the raw hydrophone and ring-array measurements is subject to institutional data policy.

## Supplementary Materials

The following supporting information can be downloaded at https://www.mdpi.com/article/10.3390/bioengineering13091070/s1, The standalone Supplementary Materials file contains Supplementary Methods S1–S6, Supplementary Results S1–S5, Tables S1–S8, and Figures S1–S19. It reports numerical convergence and cost, hydrophone processing controls, ring-water calibration and ablation, controlled FWI diagnostics, real-phantom checkpoint analyses, and the post hoc structural references.

## Abbreviations

The following abbreviations are used in this manuscript:

FDTD	Finite-difference time-domain
FWI	Full-waveform inversion
NCC	Normalized cross-correlation
RMS	Root-mean-square
RMSE	Root-mean-square error
ROI	Region of interest
USCT	Ultrasound computed tomography
