# Supplementary material for: Finite-Aperture Ultrasound Field Reproduction in Coarse-Grid FDTD via Operator-Consistent Source Reduction
Source: Bioengineering (Basel). 2026 Sep 15;13(9):1070. doi: 10.3390/bioengineering13091070 (PMC13603402; doi:10.3390/bioengineering13091070)
Supplement: Supplementary file 1 [file bioengineering-13-01070-s001.zip › bioengineering-4537359-supplementary.pdf]

bioengineering

MDPI

Article

Supplementary Materials: Finite-Aperture Ultrasound Field Reproduction in Coarse-Grid FDTD via Operator-Consistent Source Reduction

Hui Zhang †, Yang Xu †, Weicheng Yan, Yun Wu, Xiang Guo, Xiang Zhou, Pengcheng Zhang, Zaituo Li, Hongrui Tan, Junchao Zeng, Wu Qiu, Chao Cai, Mingyue Ding and Ming Yuchi *

School of Life Science and Technology, Advanced Bio-Medical Imaging Facility, Huazhong University of Science and Technology, Wuhan 430074, China; d202380973@hust.edu.cn (H.Z.); d202380996@hust.edu.cn (Y.X.); ywc@hust.edu.cn (W.Y.); clound.wu@gmail.com (Y.W.); m202472822@hust.edu.cn (X.G.); xiangzhou_@hust.edu.cn (X.Z.); m202472981@hust.edu.cn (P.Z.); lizt28@hust.edu.cn (Z.L.); d202481103@hust.edu.cn (H.T.); d202380995@hust.edu.cn (J.Z.); wuqiu@hust.edu.cn (W.Q.); chriscai@hust.edu.cn (C.C.); myding@hust.edu.cn (M.D.)

* Correspondence: m.yuchi@hust.edu.cn

† These authors contributed equally to this work.

Supplementary Methods

S1. Fine Physical Model, Convergence, and Sampling

The fine physical model represented the 2.2×13 mm^2 aperture, the cylindrical silicon lens, and the surrounding water with a variable-density scalar acoustic solver. The lens curvature was applied only in the elevation direction. Fine-grid spacings were 0.01 mm in-plane and 0.05 mm along the propagation and elevation directions. Quarter symmetry was used during the 25 μs local calculation. The fine solution was sampled at coincident target-grid nodes and target time steps before the discrete commutator was evaluated. The exterior fine physical reference used in all numerical metrics was sampled directly from the converged fine calculation rather than reconstructed from the Operator-consistent reduced source.

The target-grid comparison used isotropic 0.25, 0.50, and 0.75 mm spacings. A 0.1 Courant number was applied at every spacing, and coarse outputs were stored at 10 MHz. Field comparisons used the two orthogonal planes defined in Figure 2 of the main manuscript. Received traces were evaluated only where the fine and coarse fields shared complete temporal support.

Table S1. Numerical physical model and propagation parameters.

Section	Parameter	Value	Notes
Medium	Water sound speed / density	1500 m/s / 1000 kg/m^3	Uniform and lossless
Lens	Sound speed / density	1000 m/s / 1200 kg/m^3	20.6 mm elevation curvature radius
Transducer	Aperture	2.2×13 mm^2	Uniform normal velocity
Excitation	Pulse / band	Four-cycle 600 kHz Hann / 450–770 kHz	Peak normal velocity 1 m/s
Fine model	Spacing (x, y, z)	0.01, 0.05, 0.05 mm	25 μs local calculation
Target model	Isotropic spacing	0.25, 0.50, 0.75 mm	0.75 mm is under-resolved
Global domain	Size / duration	$100 \times 180 \times 60$ mm^3 / 180 μs	1801 stored samples
Boundary	Damping layer	6 mm	Cubic polynomial profile
Reduced source	Inner box / transition	$3 \times 4 \times 24$ mm^3 / 2 mm	Quintic smoothstep
Observation	Plane spacing	0.5 mm	Orthogonal in-plane and elevation planes

S2. Metric Definitions and Aggregation

The two-plane relative error of the root-mean-square (RMS) field was obtained by concatenating all valid samples from the in-plane and elevation fields before calculating the

normalized L_2 norm defined in the main manuscript. Spatial normalized cross-correlation (NCC) was computed separately for each plane without mean subtraction. Trace relative L_2 and trace NCC were first computed for each valid receiver and then summarized by the median. Arrival time was the peak of the analytic-signal envelope within the common direct-wave gate; signed errors were predicted minus reference arrival. Reported numerical arrival statistics used the median and 95th percentile of the absolute error.

Ring-water strength was the RMS amplitude inside one fixed 332-sample direct-wave window. Main-beam-sector relative L_2 and NCC were computed by concatenating all accepted transmitter–receiver pairs. Main-lobe axis and -3 dB width were obtained from the median angular profile. Real-phantom structural-edge cosine similarity compared the directions of smoothed image gradients inside the shared interior mask. Mean reconstruction-edge distance used the nearest post hoc structural-reference edge. Cross-acquisition NCC was computed from sound-speed updates after resampling all acquisitions to the common physical grid. Error bars over the three archived real-phantom acquisitions show population standard deviation (SD) and are descriptive rather than inferential.

S3. Operator-Consistent Reduced Source: Support, Threshold, and Cost

The primary active-support threshold retained points with temporal RMS forcing greater than 10^{-7} of the maximum. Thresholds of 10^{-8} and 10^{-6} were evaluated without changing the cutoff, target operator, or reference field. Source boxes of $3 \times 4 \times 24$, $5 \times 6 \times 24$, and $7 \times 8 \times 24$ mm^3 were evaluated with the same 2 mm transition. Storage assumed single-precision forcing histories. Runtime included only one global forward solve after loading the source package; the one-time fine calculation and source construction were reported separately.

Table S2. Numerical accuracy across target-grid spacings.

Spacing (mm)	Source representation	Two-plane RMS-field relative L_2	In-plane NCC	Elevation NCC	Median trace L_2	Arrival P95 (μs)
0.25	Point source	1.21125	0.92424	0.74517	1.28774	1.8
0.25	Direct coarse-grid aperture source	0.16307	0.99228	0.98421	0.88307	1.9
0.25	Operator-consistent reduced source	0.00615	1.00000	0.99996	0.01937	0.1
0.50	Point source	1.21117	0.92423	0.74509	1.30740	1.8
0.50	Direct coarse-grid aperture source	0.16414	0.99294	0.98431	0.90330	1.9
0.50	Operator-consistent reduced source	0.00583	1.00000	0.99996	0.03691	0.1
0.75	Point source	0.90060	0.90498	0.72497	0.98261	2.5
0.75	Direct coarse-grid aperture source	0.33931	0.97203	0.96026	0.80038	2.2
0.75	Operator-consistent reduced source	0.30073	0.97967	0.97546	0.73260	2.2

Table S3. Support and cost sensitivity of the Operator-consistent reduced source on the 0.50 mm target grid.

Sweep	Setting	Active nodes	Storage (MiB)	Two-plane RMS-field relative L_2	Forward runtime (s)
Threshold	10^{-8}	36,219	179.61	0.00583236	64.98
Threshold	10^{-7}	32,619	161.76	0.00583233	65.05
Threshold	10^{-6}	28,709	142.37	0.00583234	65.13
Source box	$3 \times 4 \times 24$ mm^3	32,619	161.76	0.00548176	67.17
Source box	$5 \times 6 \times 24$ mm^3	47,073	233.44	0.00543716	64.94
Source box	$7 \times 8 \times 24$ mm^3	63,648	315.64	0.00541863	64.56

S4. Hydrophone Processing and Registration

The hydrophone comparison used the native measurement sampling and the 450–770 kHz analysis band. Numerical RMS fields were linearly interpolated to the hydrophone coordinates. Each measured and numerical plane was independently peak-normalized; no absolute pressure calibration was claimed. A global axial shift was preregistered by examining the feasible full-overlap interval from 0 to 10 mm and then frozen at +10 mm. The same shift was applied to every source representation and both planes. No field was laterally stretched or method-specifically translated. Additive offsets used in Figure 7 profiles were display-only quantities and were not used in Table S4.

https://doi.org/10.3390/bioengineering13091070

Table S4. Hydrophone field metrics and numerical reduction fidelity.

Reference	Plane	Point source L_2/NCC	Direct coarse-grid aperture source L_2/NCC	Operator-consistent reduced source L_2/NCC	Fine physical reference L_2/NCC
Hydrophone	In-plane	0.6375 / 0.9701	0.6031 / 0.9440	0.4912 / 0.9350	0.4899 / 0.9354
Hydrophone	Elevation	0.6165 / 0.8949	0.1146 / 0.9944	0.2438 / 0.9828	0.2434 / 0.9828
Fine physical model	In-plane	0.3487 / 0.9708	0.1498 / 0.9984	0.00240 / 1.0000	—
Fine physical model	Elevation	0.9580 / 0.8265	0.3084 / 0.9890	0.00215 / 1.0000	—

S5. Ring-Water Calibration Controls

The 110 mm ring radius and the single 262-sample delay convention were fixed before fitting. Water sound speed was estimated from water1, and the aggregate signed-arrival residual was examined only to verify the geometry–timing convention. The common waveform was cropped from the unfiltered opposite-channel trace before band limitation. The primary comparison used the 3.8 mm effective acoustic aperture selected using the water1 main-lobe width. The 2.2 mm aperture of the frozen physical model was retained as the prespecified nominal-aperture sensitivity condition. Per-transmit strength and pointing corrections were fitted only within the observed −3 dB main lobe, and water2/water3 were never used to update these parameters.

Table S5. Blind ring-water results averaged over water2 and water3 for the fixed 2.2 mm nominal-aperture sensitivity condition.

Source representation	Strength relative L_2	Strength NCC	Median absolute error (dB)	Beam-axis error (deg)	Width error (deg)
Point source	0.48617	0.93171	1.8272	39.0	—
Direct coarse-grid aperture source	0.27404	0.97273	1.2630	1.0	39.68
Operator-consistent reduced source	0.23804	0.97827	1.1262	1.0	27.07

S6. Controlled FWI and Real-Phantom FWI Diagnostics

The controlled inversion used accepted line-search steps only. A zero step indicated that no tested step decreased the objective, after which the method was stopped. In the real-phantom experiments, all methods were instead reported at the prespecified iteration 200. Before each real-phantom inversion, the water-derived waveform, water sound speed, and applicable per-transmit calibration parameters were loaded from the corresponding source package. For each source representation, one additional scalar was estimated from all selected phantom traces under the homogeneous initial model using the RMS-matching expression reported in the main Methods; this scalar was then fixed for the complete 200-iteration run. No receiver-wise gain, trace-wise time shift, or iteration-dependent source update was used. Training-loss and model-update histories were used to assess stability. They were not held-out curves and were not used to select separate endpoints. The 3 MHz structural sections were registered after inversion, and the resulting cylinder masks were fixed across methods within each acquisition.

The three result archives were identified by the acquisition operator as phantom acquisitions 1–3 and were verified by distinct SHA-256 hashes. For acquisitions 2 and 3, runtime object-file overrides were not persisted back into the static notebook source cells or YAML snapshots; the archive manifests therefore provide the authoritative data labels and explicitly record this provenance limitation. Resolved runtime snapshots should be retained in any subsequent confirmatory acquisition.

Table S6. Controlled FWI terminal outcomes. RMSE denotes root-mean-square error; MAE denotes mean absolute error.

Source representation	RMSE (m/s)	MAE (m/s)	Model relative L_2	Model correlation	Final data loss
Initial model	8.2576	2.7134	1.0000	0.0000	—
Point source	4.9429	3.6842	0.5986	0.8830	0.05616
Direct coarse-grid aperture source	5.1697	4.3417	0.6261	0.9199	0.01868
Operator-consistent reduced source	2.1362	1.1543	0.2587	0.9686	0.00630

https://doi.org/10.3390/bioengineering13091070

Table S7. Real-phantom outcomes for each repeated acquisition.

Acquisition	Source representation	Training loss	Structural-edge cosine similarity	Mean reconstruction-edge distance (mm)	RMS change, iterations 160–200 (m/s)	Interior median (m/s)
1	Point source	0.74242	0.54391	4.356	2.278	1522.32
1	Direct coarse-grid aperture source	0.84732	0.54701	5.222	1.714	1519.46
1	Operator-consistent reduced source	0.85199	0.53513	8.136	2.218	1524.08
2	Point source	0.74362	0.48457	4.020	2.322	1522.23
2	Direct coarse-grid aperture source	0.84788	0.54092	4.373	1.711	1519.52
2	Operator-consistent reduced source	0.85248	0.58181	5.940	2.179	1523.99
3	Point source	0.74422	0.49215	4.209	2.285	1522.39
3	Direct coarse-grid aperture source	0.84714	0.55168	4.662	1.763	1519.23
3	Operator-consistent reduced source	0.85141	0.58404	6.045	2.271	1523.48

Table S8. Cross-acquisition repeatability of reconstructed sound-speed updates.

Source representation	Pairwise update RMSE (m/s), mean $\pm$ SD	Pairwise update NCC, mean $\pm$ SD
Point source	9.18 ± 0.10	0.9431 ± 0.0015
Direct coarse-grid aperture source	9.66 ± 0.16	0.8729 ± 0.0047
Operator-consistent reduced source	13.90 ± 0.10	0.7160 ± 0.0037

Supplementary Results

S1. Wavefield Evolution and Source-Support Sensitivity

The instantaneous fields showed that the Point source generated the wrong elevation curvature from the near field onward, whereas the Direct coarse-grid aperture source captured the broad field but retained phase differences. The Operator-consistent reduced source followed the fine physical reference through 80 μs on both planes (Figures S1 and S2). Increasing the source box changed the exterior metrics only marginally, while active-node count and storage increased substantially (Figures S3–S5). This result indicates that the transition layer already enclosed the device-scale field needed by the target operator.

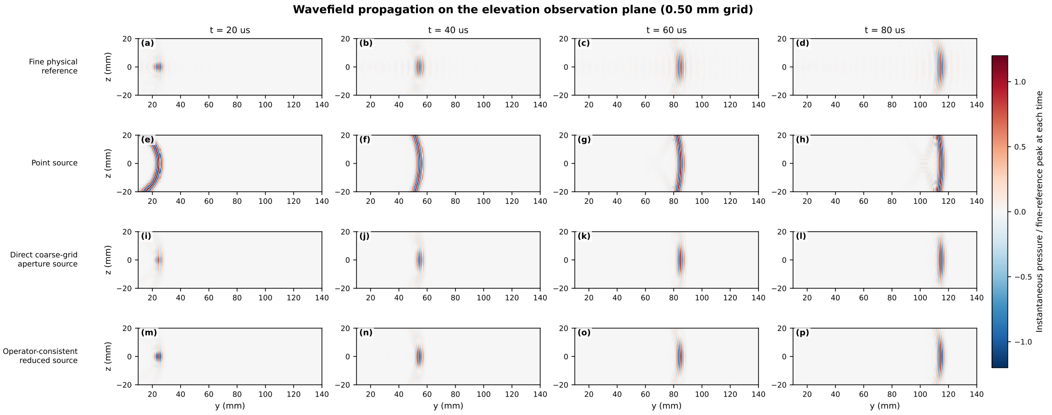

Figure S1. Elevation-plane instantaneous wavefield propagation. Columns show 20, 40, 60, and 80 μs; rows show the Fine physical reference, Point source, Direct coarse-grid aperture source, and Operator-consistent reduced source. Each column is normalized by the Fine physical reference peak at that time and displayed at the true physical aspect ratio.

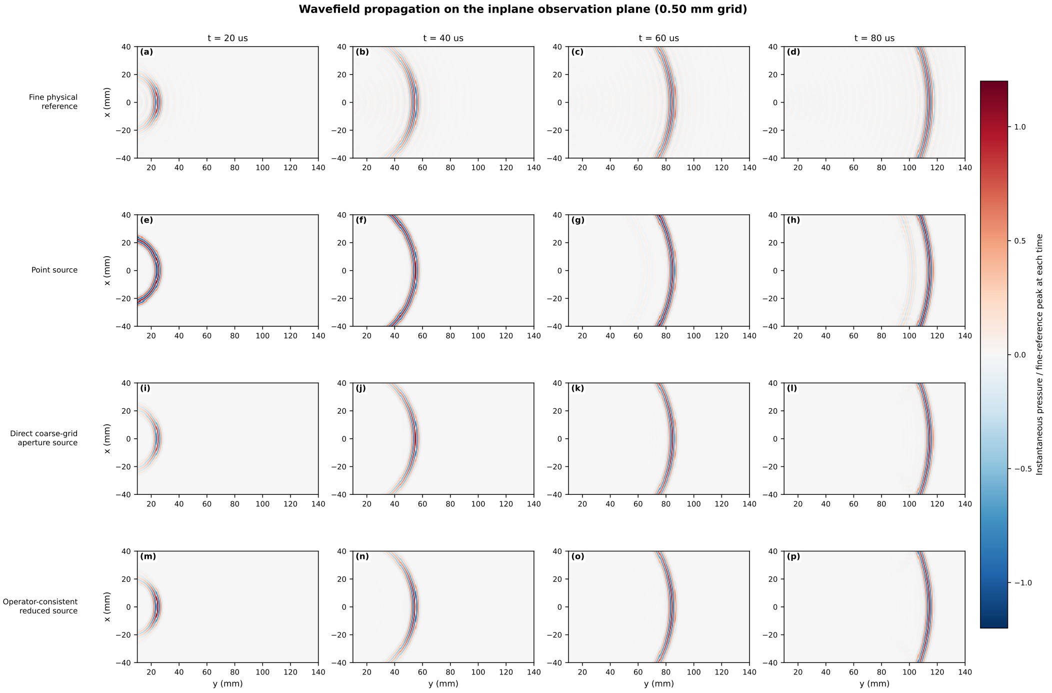

Figure S2. In-plane instantaneous wavefield propagation using the normalization and display rules of Figure S1.

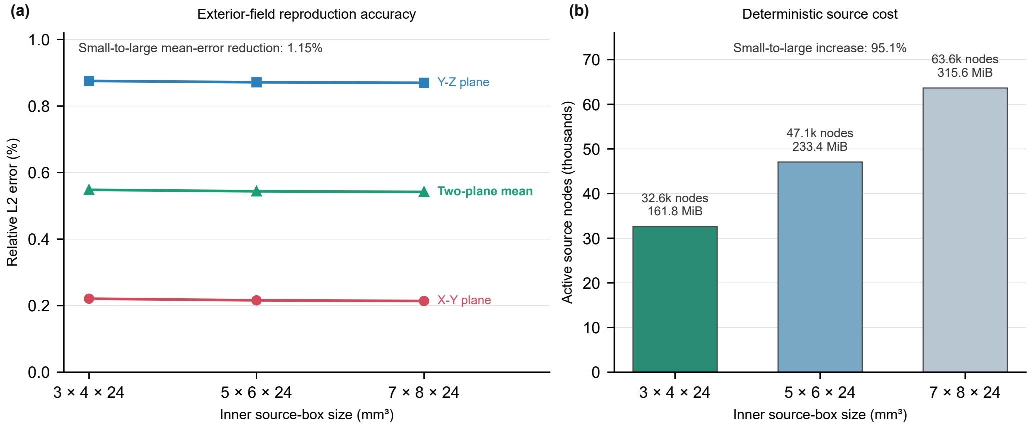

Figure S3. Source-box convergence and deterministic source cost for $3 \times 4 \times 24$, $5 \times 6 \times 24$, and $7 \times 8 \times 24$ mm^3 inner boxes.

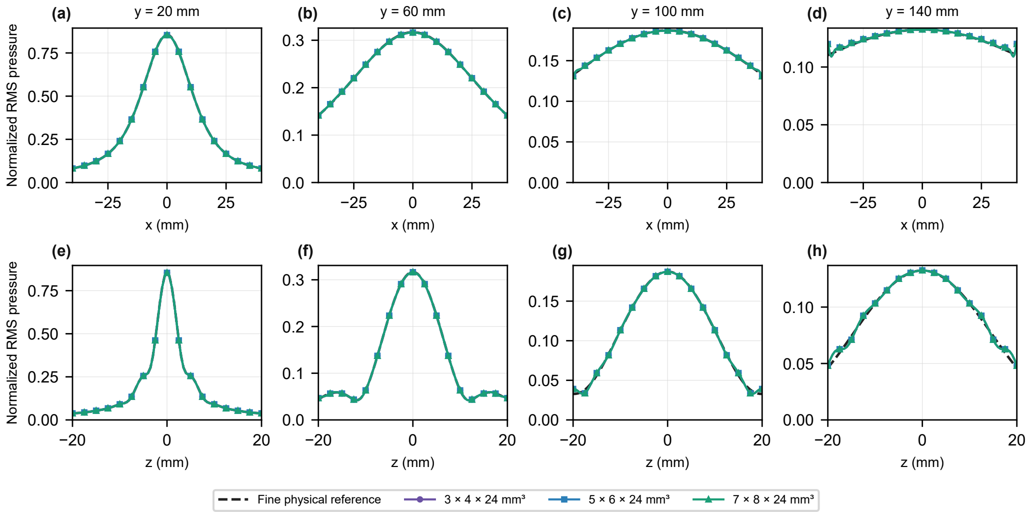

Figure S4. Source-box profile comparison at propagation distances of 20, 60, 100, and 140 mm.

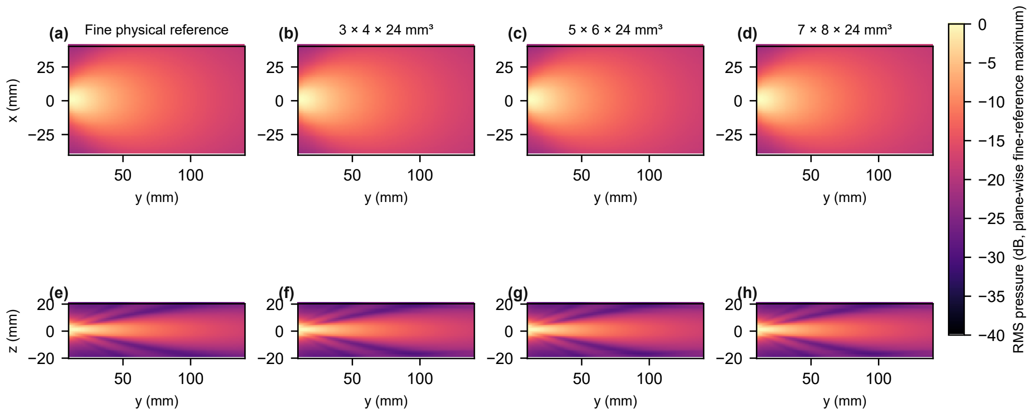

Figure S5. Orthogonal RMS fields for the Fine physical reference and three source-box sizes on common dB scales.

S2. Hydrophone Profiles Were Consistent with a Residual Physical-Model Gap

The preregistered coordinate search supported the +10 mm global correction without method-specific alignment. After this correction was fixed, the Operator-consistent reduced source remained close to the fine physical reference, although it did not yield the lowest error in every hydrophone comparison. The hydrophone results therefore separate source-reduction error from residual error in the physical transducer model.

S3. Ring-Water Controls and Effective-Aperture Ablation

Water sound speed, waveform, and timing diagnostics were stable across the three acquisitions (Figure S6). Blind angular maps and profiles were visually repeatable (Figures S7 and S8). At the fixed 2.2 mm aperture, blind relative L_2 errors were approximately 0.274 for the Direct coarse-grid aperture source and 0.238 for the Operator-consistent reduced source. Selecting the 3.8 mm effective aperture on water1 lowered them to 0.147 and 0.153, respectively, while the Operator-consistent reduced source retained the smaller main-lobe-width error (Figure S12). This ablation shows that the effective aperture addresses a shared physical-model width mismatch; it does not replace the comparison between target-grid source representations.

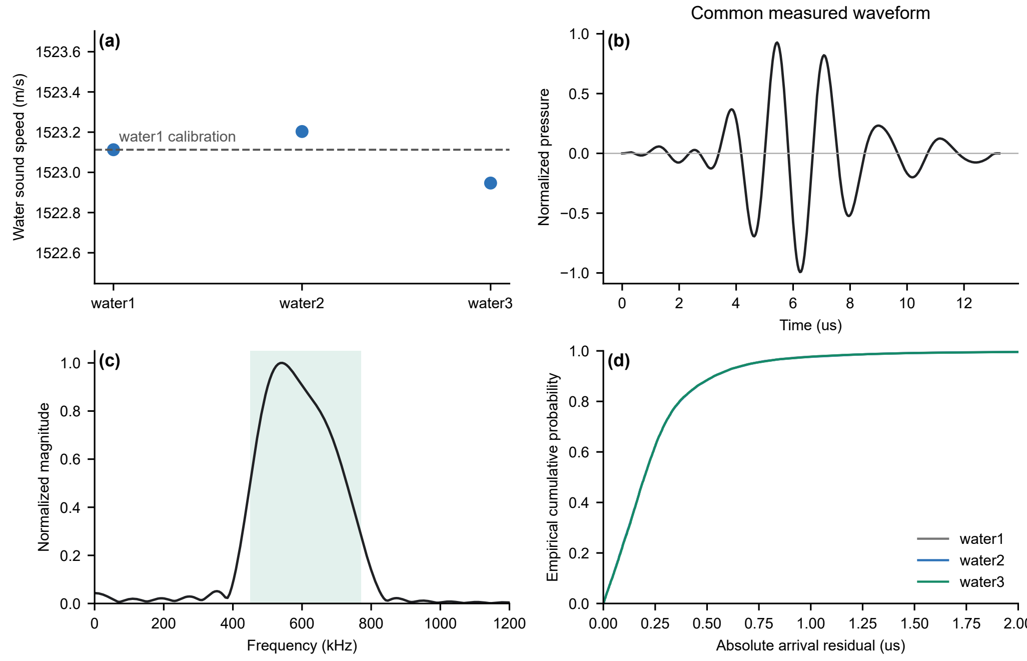

Figure S6. Ring-water calibration controls: water sound speed estimates, common measured waveform, analysis-band spectrum, and aggregate signed arrival-time residual.

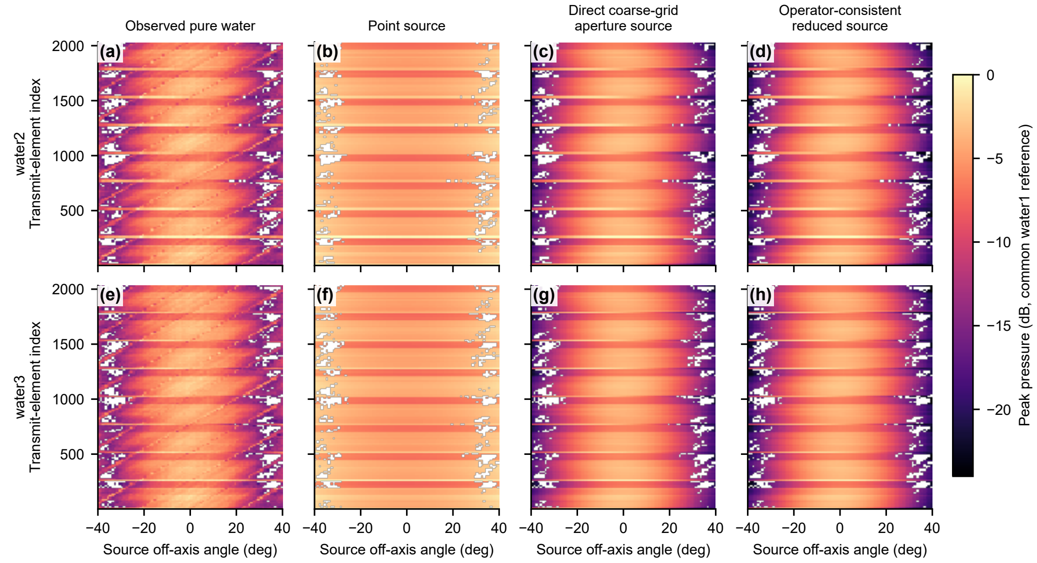

Figure S7. Blind ring-water angular-strength maps for water2 and water3 under a common normalization and angular window.

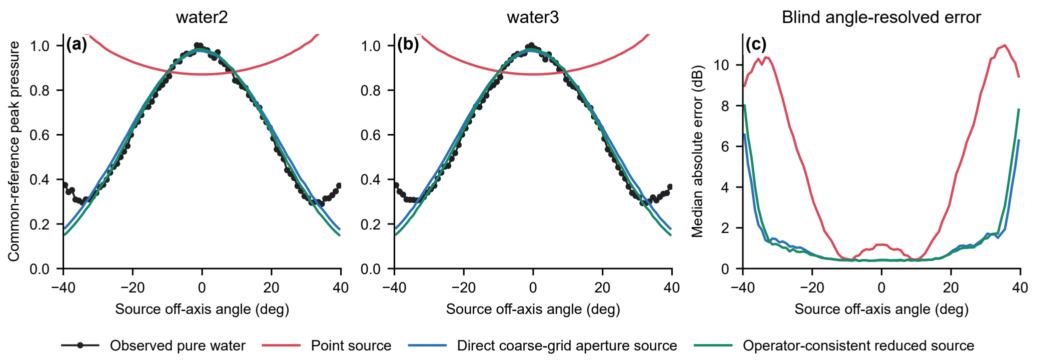

Figure S8. Blind median angular profiles and angle-resolved absolute errors for water2 and water3.

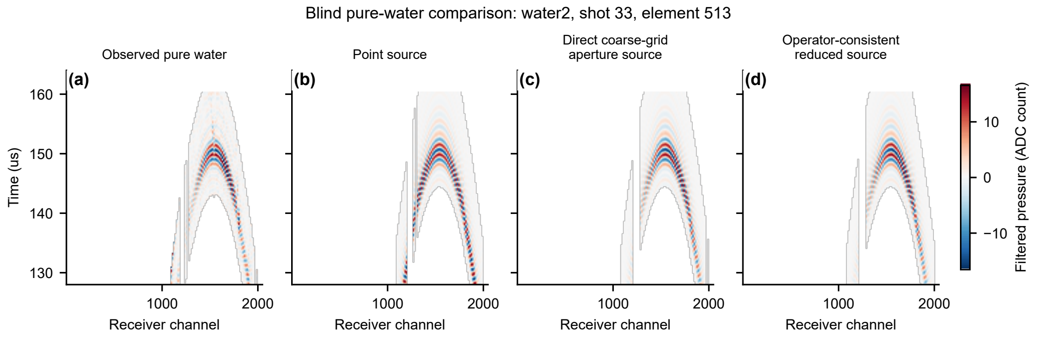

Figure S9. Representative blind time–channel comparison for water2, shot 33, over the same receiver sector and time interval.

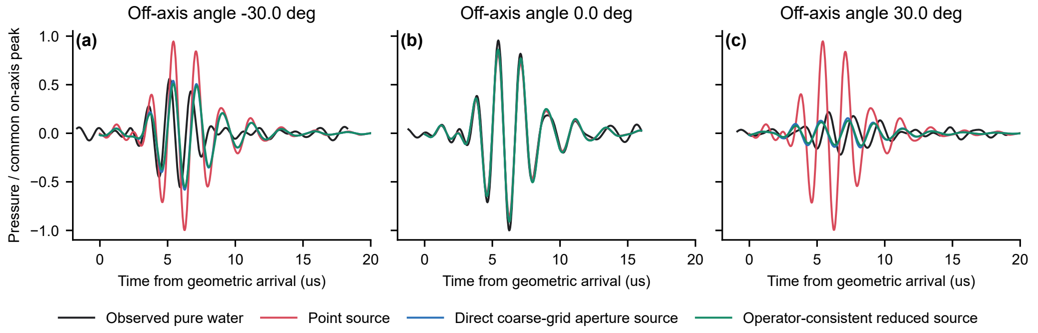

Figure S10. Representative blind water2 waveforms at −30, 0, and +30 degrees without channel-wise normalization or per-trace shifting.

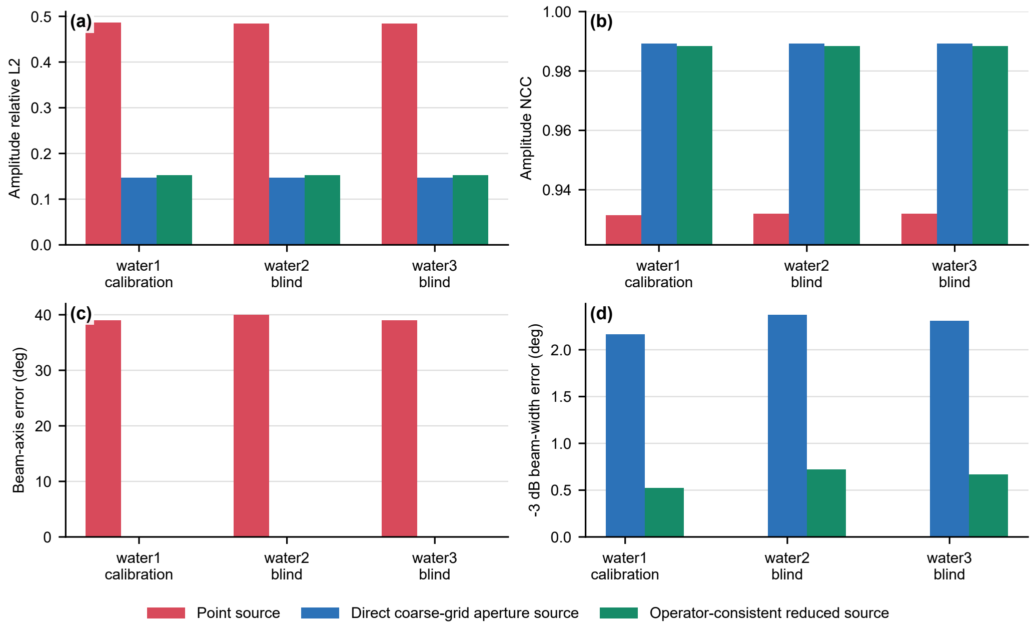

Figure S11. Ring-water strength, NCC, beam-axis, and −3 dB main-lobe-width metrics for calibration and blind acquisitions.

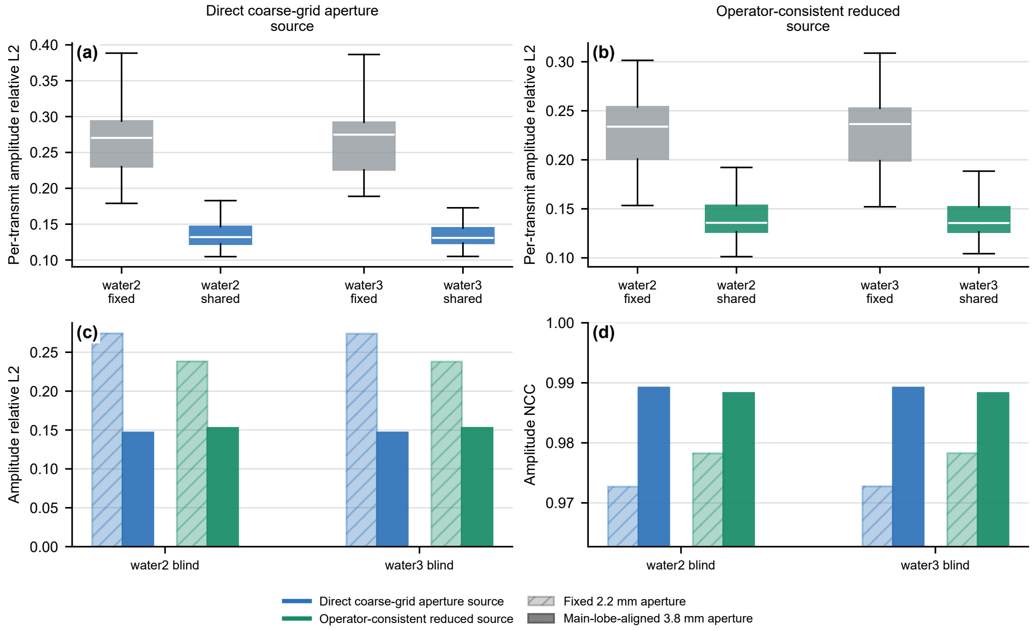

Figure S12. Effective-aperture ablation comparing the fixed 2.2 mm aperture with the water1-selected 3.8 mm effective aperture on both blind acquisitions.

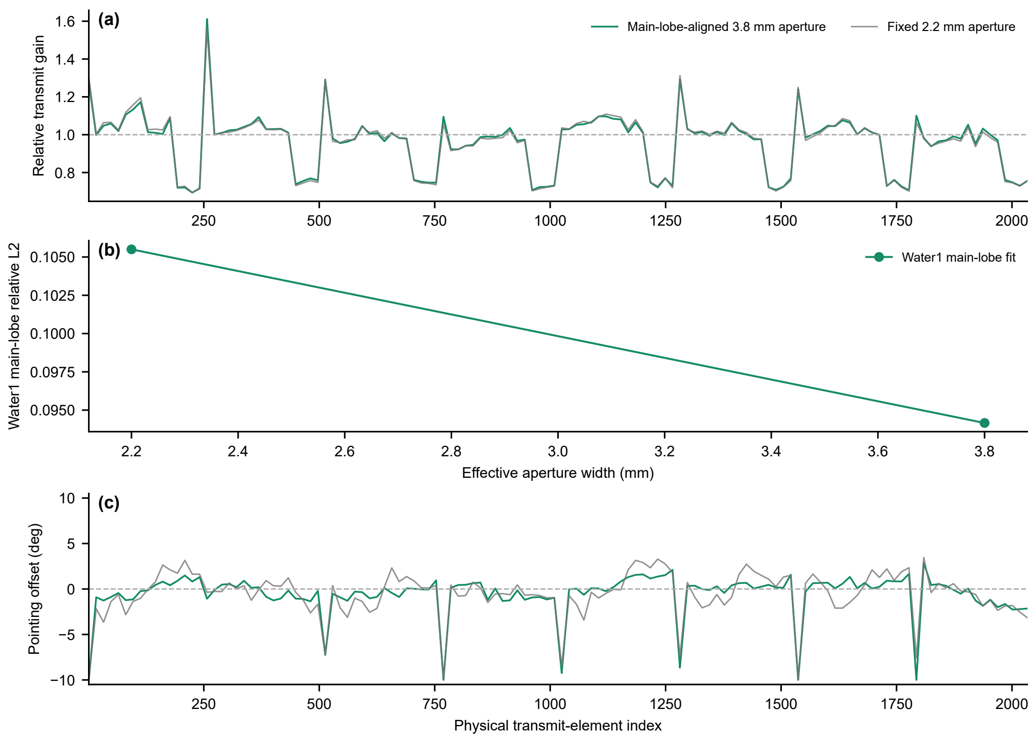

Figure S13. Per-transmit relative strength, effective-aperture sensitivity, and fitted pointing offsets under the frozen geometry and timing convention.

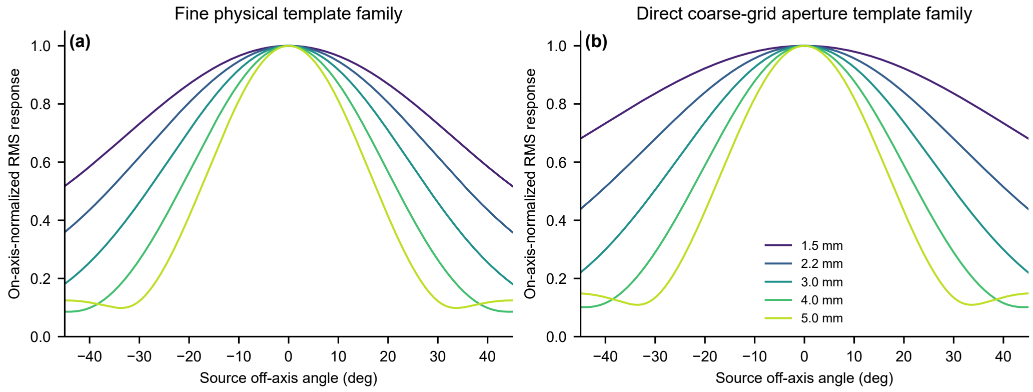

Figure S14. Normalized angular templates used to examine effective-aperture sensitivity for the Fine physical model and Direct coarse-grid aperture source.

S4. Controlled FWI Target Recovery and Convergence

Target-centred profiles showed that all methods recovered the sign and position of the three inclusions, but the Operator-consistent reduced source most closely followed their interior levels and transition widths (Figure S15). Accepted-loss curves decreased monotonically by construction, whereas true-model RMSE did not decrease monotonically for the source-mismatched baselines (Figure S16). This divergence confirms that data loss alone is insufficient to rank controlled reconstructions when the forward source is wrong.

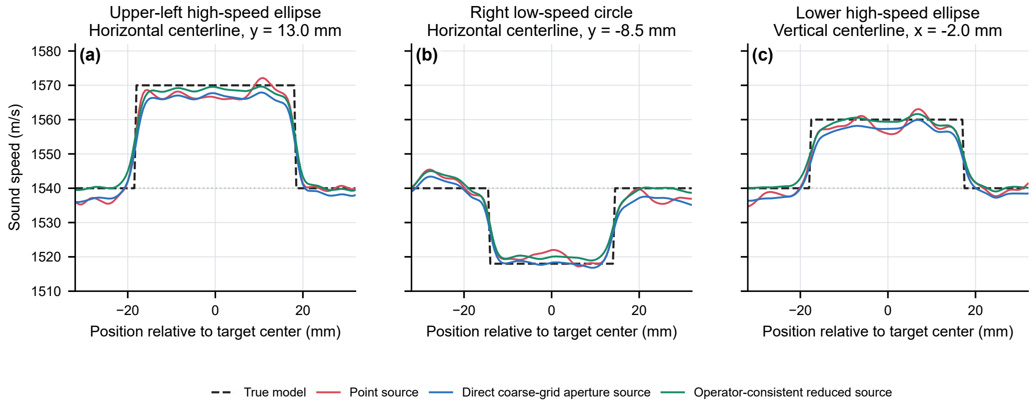

Figure S15. Native-grid centre lines through the three controlled FWI targets. No smoothing or local normalization is applied.

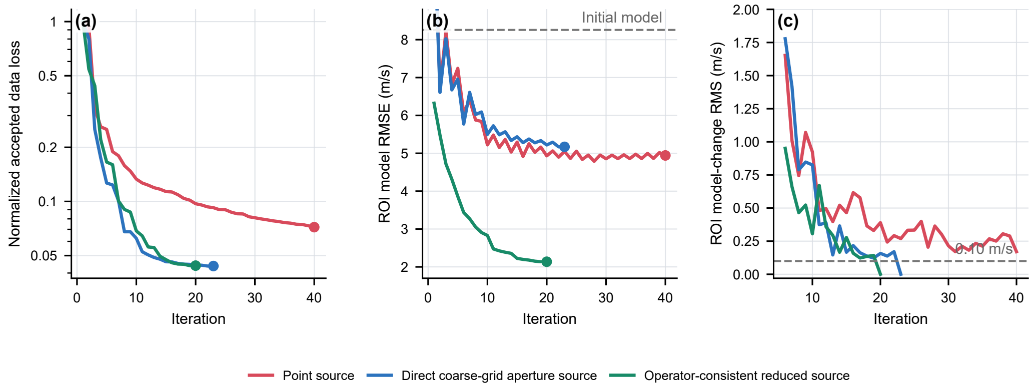

Figure S16. Accepted normalized data loss, true-model region-of-interest (ROI) RMSE, and RMS model change. True-model RMSE is post hoc and was not used for stopping.

S5. Real-Phantom Late Evolution and Structural Reference

Checkpoint maps showed continuing but spatially coherent updates from iteration 80 to 200 for every method (Figure S17). The iteration-160-to-200 NCC exceeded 0.996 for all nine reconstructions, although nonzero RMS change remained (Figure S18). Reporting the common iteration-200 endpoint therefore avoids method-specific selection from training histories. The structural references and fixed cylinder registrations are shown in Figure S19; neither contributed to the optimization.

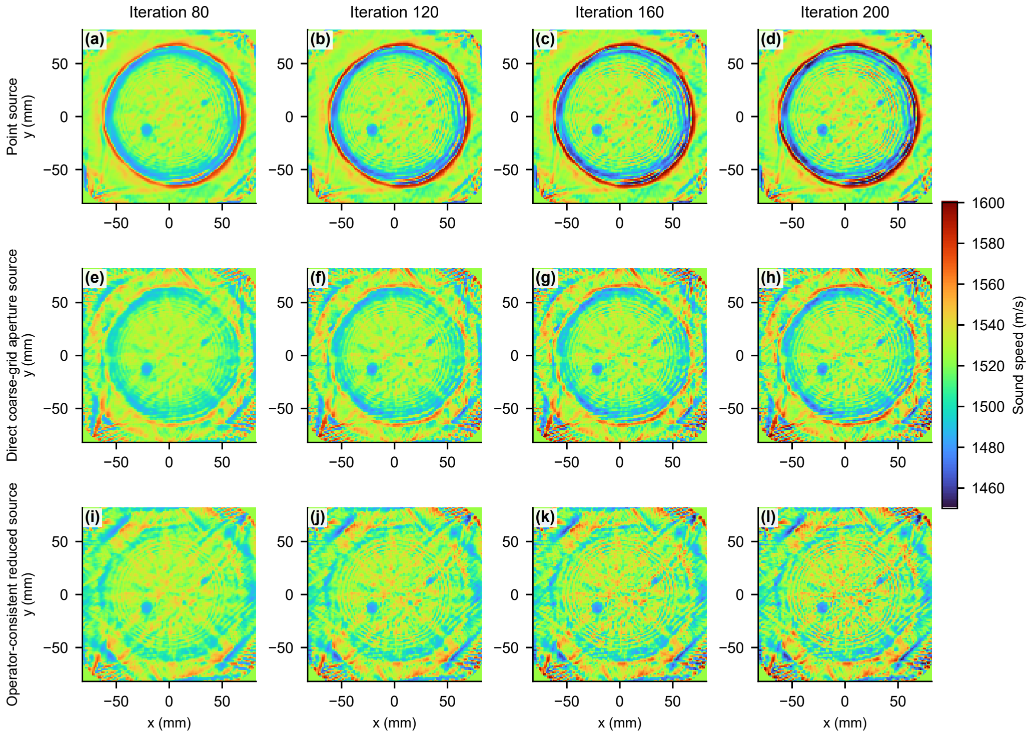

Figure S17. Representative real-phantom maps at iterations 80, 120, 160, and 200 for all three source representations on a shared sound-speed scale.

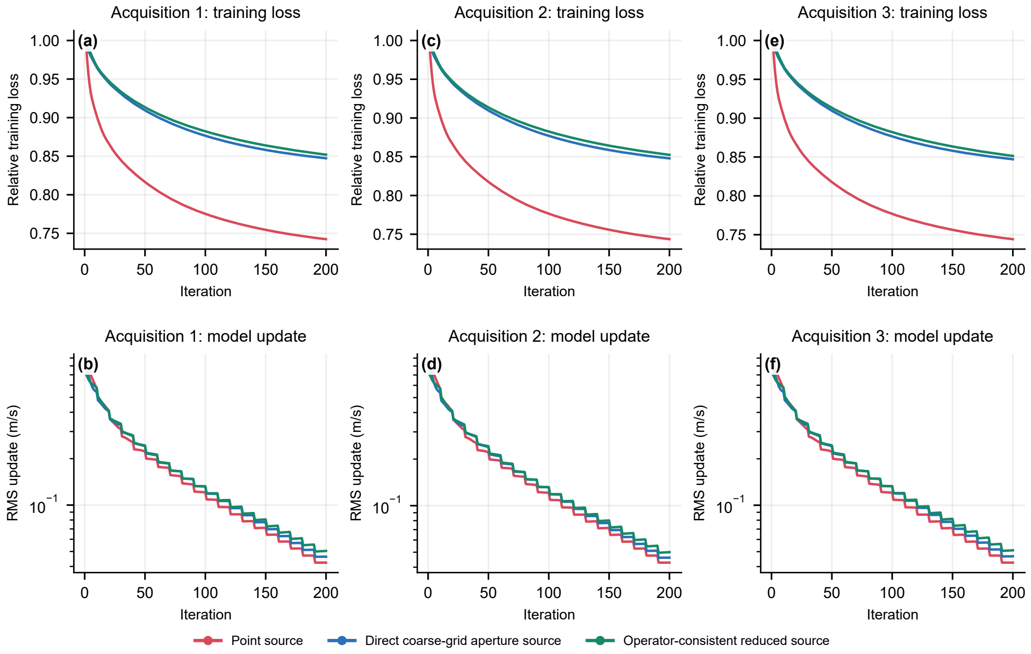

Figure S18. Real-phantom training-loss and RMS model-update histories for all acquisitions and source representations. These histories assess numerical stability and are not held-out validation curves.

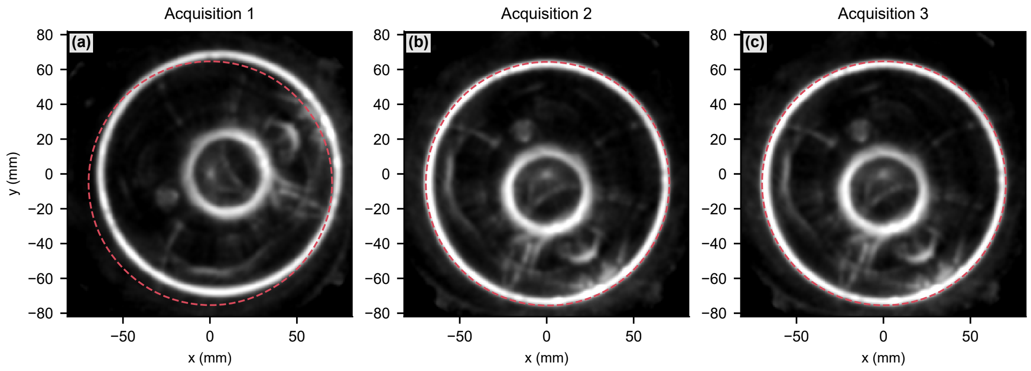

Figure S19. Post hoc 3 MHz structural sections and fixed cylinder geometry used to define the common interior evaluation mask. The images were not used for FWI, source calibration, or method-specific tuning.
